# Several *N*-Glycans on the HIV Envelope Glycoprotein gp120 Preferentially Locate Near Disulphide Bridges and Are Required for Efficient Infectivity and Virus Transmission

**DOI:** 10.1371/journal.pone.0130621

**Published:** 2015-06-29

**Authors:** Leen Mathys, Jan Balzarini

**Affiliations:** Rega Institute for Medical Research, KU Leuven, Leuven, Belgium; Institute of Infection and Global Health, UNITED KINGDOM

## Abstract

The HIV envelope glycoprotein gp120 contains nine disulphide bridges and is highly glycosylated, carrying on average 24 *N*-linked glycans. Using a probability calculation, we here demonstrate that there is a co-localization of disulphide bridges and *N*-linked glycans in HIV-1 gp120, with a predominance of *N*-linked glycans in close proximity to disulphide bridges, at the C-terminal side of the involved cysteines. Also, *N*-glycans are frequently found immediately adjacent to disulphide bridges in gp120 at the N-terminal side of the involved cysteines. In contrast, *N*-glycans at positions close to, but not immediately neighboring disulphide bridges seem to be disfavored at the N-terminal side of the involved cysteines. Such a pronounced co-localization of disulphide bridges and *N*-glycans was also found for the *N*-glycans on glycoprotein E1 of the hepatitis C virus (HCV) but not for other heavily glycosylated proteins such as E2 from HCV and the surface GP from Ebola virus. The potential functional role of the presence of *N*-glycans near disulphide bridges in HIV-1 gp120 was studied using site-directed mutagenesis, either by deleting conserved *N*-glycans or by inserting new *N*-glycosylation sites near disulphide bridges. The generated HIV-1_NL4.3_ mutants were subjected to an array of assays, determining the envelope glycoprotein levels in mutant viral particles, their infectivity and the capture and transmission efficiencies of mutant virus particles by DC-SIGN. Three *N*-glycans located nearby disulphide bridges were found to be crucial for the preservation of several of these functions of gp120. In addition, introduction of new *N*-glycans upstream of several disulphide bridges, at locations where there was a significant absence of *N*-glycans in a broad variety of virus strains, was found to result in a complete loss of viral infectivity. It was shown that the *N*-glycan environment around well-defined disulphide bridges of gp120 is highly critical to allow efficient viral infection and transmission.

## Introduction

The envelope of the human immunodeficiency virus (HIV) carries two virus-encoded glycoproteins: the surface gp120 and the non-covalently associated transmembrane gp41. The former is highly glycosylated, carrying 18 to up to 32 *N*-linked glycans (24 on average) which occur clustered on the protein and constitute about 50% of the molecular weight of gp120 [1–3]. Although still subject of discussion, it is nowadays assumed that at least 56–73% of these *N*-linked glycans on gp120 are high-mannose-type glycans [4], which is unusual as compared to cellular glycoproteins containing mostly, if not exclusively, complex-type glycans [5]. In addition to *N*-glycans, gp120 is also characterized by 18 cysteines which form 9 disulphide bridges. It has been shown that 5 disulphide bridges are crucial for the correct folding of gp120 and six or seven of the disulphide bridges in gp120 are found to be indispensable for appropriate envelope function [6].

During glycoprotein biosynthesis, *N*-glycosylation and disulphide bridge formation in the endoplasmic reticulum (ER) are considered to have a reciprocal influence on each other. *N*-glycans may sterically hinder the formation of disulphide bridges [7] and disulphide bridges in turn may prevent the use of a nearby *N*-glycosylation site [8]. Normally, the addition of *N*-linked glycans to the asparagine of an *N*-glycosylation motif (Asn-X-Thr/Ser, in which X is not proline) by the ER oligosacharyl transferase in the nascent protein backbone initiates the recognition of glycoproteins by the ER resident lectins calreticulin and calnexin. These lectins act as chaperones and enhance folding of glycoproteins by the recruitment and activation of the disulphide isomerase ERp57, which is responsible for the formation of disulphide bridges in glycoproteins (reviewed in [9]). Together, calnexin, calreticulin and ERp57 are responsible for the correct folding of the HIV precursor envelope glycoprotein gp160 in the ER, after which it is cleaved to gp120 and gp41 in the Golgi apparatus (reviewed in [10]).

**Fig 1:**
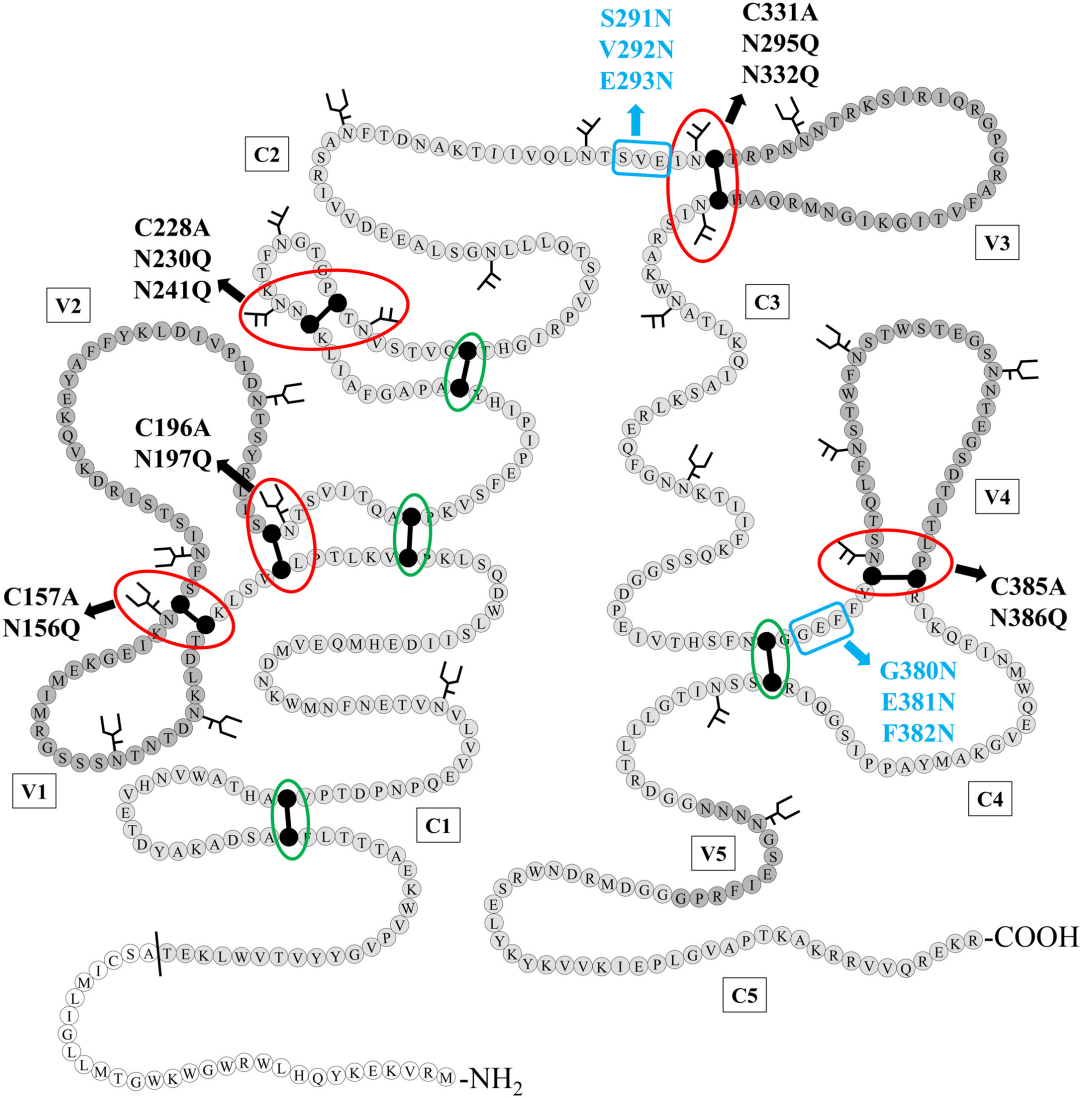
Schematic structure of HIV-1_NL4.3_ gp120 with indication of the *N*-glycans and disulphide bridges that were deleted/inserted using site-directed mutagenesis. Disulphide bridges that were found in close proximity to one or two *N*-glycosylation sites, either directly neighboring or separated from each other by one other amino acid, are indicated with a red circle. Disulphide bridges that are not directly neighbored by *N*-glycans are indicated by a green circle. Mutations resulting in deleted disulphide bridges (Cys→Ala) or *N*-glycosylation sites (Asn→Gln) are indicated in black. Mutations resulting in the generation of novel *N*-glycosylation sites, due to the introduction of an Asn-X-Ser motif, are indicated in blue (only the glycosylated Asn is shown). The N-terminal signal sequence is colored in white, the conserved domaines (C1–C5) in light grey and the variable loops (V1–V5) in dark grey. Cysteines that are involved in a disulphide bridge are colored black. Figure based on Leonard *et al*. [3]. Amino acid numbering according to HIV-1 strain HXB2.

By inspecting the structure of HIV-1_NL4.3_ gp120, we noticed that more than half of the disulphide bridges are immediately surrounded by *N*-linked glycans (Fig 1). These observations may suggest that the presence of *N*-glycans immediately near, or close to the disulphide bridges in gp120 is not a coincidence and that there might exist a specific functional interference between the location of disulphide bridges and these *N*-linked glycosylation sites in HIV gp120.

Given the important role of disulphide bridges and *N*-glycans during glycoprotein biosynthesis, we wondered whether neighboring disulphide bridges and *N*-glycans could have a central, indispensable role in gp120 biosynthesis and functionality. To address this issue, the distribution and appearance of neighboring disulphide bridges and *N*-glycans in gp120 was first investigated using an alignment of a wide variety of different HIV-1 subtypes. Next, a broad variety of mutant HIV-1_NL4.3_ strains lacking disulphide bridges and/or the neighboring *N*-glycans in gp120 was generated. In addition, new *N*-glycosylation sites were introduced in HIV-1_NL4.3_ gp120 at positions that were found to be disfavored for glycosylation in wild-type (WT) gp120. We examined the influence of these mutations on the biosynthesis and functionality of gp120 to explore the importance of neighboring disulphide bridges and *N*-glycans in HIV-1 gp120.

## Materials and Methods

### Probability calculations for co-localization of disulphide bridges and *N*-glycosylation sites in HIV gp120

The position, number and level of conservation of disulphide bond-engaged cysteines in HIV-1 gp120 was determined. Therefore, the HIV Env sequence compendium (2014) available on the HIV sequence database website [11] was consulted. The level of conservation of the disulphide bond-engaged cysteines was determined by calculating the occurrence of these cysteines in 180 HIV-1 sequences of HIV-1 strains belonging to group M (including group M recombinants) [11]. In total, 18 disulphide bond-engaged cysteines were found in HIV-1 gp120 and all were at least 98% conserved (S1 Table).

Next, the positions of *N*-linked glycosylation sites in HIV-1 gp120 were determined using the N-glycosite software available on the HIV sequence database website [11], based on an alignment of consensus sequences (2004) [11]. The alignment contained consensus sequences for HIV-1 subtypes A1, A2, B, C, D, F1, F2, G, H, CRF01-AE, CRF02-AG, CRF03-AB, CRF04-cpx, CRF06-cpx, CRF08-BC, CRF10-CD, CRF11-cpx, CRF12-BF, and CRF14-BG. The consensus of consensus sequences and the ancestral sequences were excluded from the analysis. For the calculations, a distinction was made between *N*-glycosylation sites with at least 50% conservation (24 *N*-glycosylation sites) and *N*-glycosylation sites with less than 50% conservation (33 *N*-glycosylation sites). The frequency of the actual occurrence of conserved and non-conserved *N*-glycans at a distance of 1, 2, 3, 4 or 5 amino acids away from a cysteine involved in a disulphide bridge was calculated and compared to the frequency that could be expected in case of random distribution of 24 conserved and 33 non-conserved *N*-glycans across gp120. We made a distinction between the *N*-glycans at the N-terminal side and at the C-terminal side of the cysteine. For a more elaborate clarification of these calculations, the supplementary S2 Table should be consulted.

Similar calculations were also performed for the envelope glycoproteins of Ebola virus and hepatitis C virus (HCV). Sequence information was obtained from the NCBI database [12]: UniProtKB codes (Q05320.1) and (O11457.1) for Ebola GP; GenBank ID (ABC40379.1) for HCV E1 and NCBI Reference Sequence (NP_751921.1) for HCV E2. The allocation of *N*-glycosylation sites and disulphide bridges in GP, E1 and E2 was based on publications of Jeffers *et al*. [13], Wahid *et al*. [14] and Krey *et al*. [15] respectively.

### Cells

Human embryonic kidney cells (HEK293T) were obtained from the American Type Culture Collection (ATCC) (Manassas, VA) and were grown in Dulbecco’s Modified Eagle Medium (DMEM) (Invitrogen, Merelbeke, Belgium), supplemented with 10% fetal calf serum (FCS) (Sigma, Bornem, Belgium), 75 mM NaHCO_3_ and 2% gentamicin (Invitrogen).

Human CD4^+^ T lymphocytic C8166 cells were obtained from ATCC. Human B lymphocytic Raji/DC-SIGN cells, expressing dendritic cell-specific intercellular adhesion molecule-3-grabbing non-integrin (DC-SIGN), were constructed by Geijtebeek *et al*. [16] and kindly provided by Dr. L. Burleigh (Institut Pasteur, Paris, France). These cell lines were both grown in RPMI-1640 medium (Invitrogen), supplemented with 10% FCS (Sigma), 75 mM NaHCO_3_, 2 mM L-glutamine and 2% gentamicin (Invitrogen).

### Site-directed mutagenesis

Mutations were introduced into the plasmid pBlue_Env [17], encoding the precursor envelope glycoprotein gp160 of HIV-1_NL4.3_. Mutagenesis was achieved using the Quikchange Site-Directed Mutagenesis Kit (Agilent Technologies, Diegem, Belgium) and the primers listed in S3 Table. Plasmid DNA was purified with the PureLink Quick Plasmid Miniprep Kit (Invitrogen) and sequenced with the ABI PRISM BigDye Terminator v3.1 Ready Reaction Cycle Sequencing Kit (Applied Biosystems, Ghent, Belgium) to confirm the presence and identity of the desired mutations. The obtained amplicons were loaded onto the ABI3100 Genetic Analyzer (Applera, Nieuwekerk a/d Issel, The Netherlands) after which the sequences were analyzed using the Geneious Pro 5.5.6 software.

The oligonucleotide sequences of all primers, of which some have been published before [18], are listed in S3 Table. The different mutations that were introduced into HIV-1_NL4.3_ gp120 are presented in Fig 1 and listed in S4 Table.

### Viruses

HIV-1_NL4.3_ was recombinantly produced as described previously [19]. Briefly, HEK293T cells were cotransfected with a PCR product encoding gp160 and pNL4.3_ΔEnv_eGFP linearized by XbaI-digestion. This results in homologous recombination, inserting the gp160 gene in the pNL4.3_eGFP backbone. Therefore, transfected and infected cells express enhanced green fluorescent protein (eGFP). The construct pNL4.3_ΔEnv_eGFP was kindly provided by Dr. M.E. Quiñones‐Mateu (Lerner Research Institute, Cleveland, OH, USA) [20].

HIV-1_NL4.3_ lacking envelope glycoproteins was produced by transfecting the construct pNLHIVxΔUΔss into HeLa-*tat*-III cells using GeneCellIn (BioCellChallenge, Nivelles, Belgium), according to the instructions of the manufacturer. The construct pNLHIVxΔUΔss, was a kind gift from Dr. Alenka Jejcic (at that time at the Karolinska Institute, Stockholm, Sweden).

### Western blotting to evaluate Env incorporation in viral particles

To determine the expression and subsequent incorporation of the envelope glycoproteins gp120 and gp41 in viral particles, virus concentrates equaling 50 ng of p24 capsid protein were loaded onto a 4–12% Bis-Tris PAGE gel (Invitrogen). After blotting to a PVDF membrane (GE healthcare life sciences, Diegem, Belgium), gp120, gp41 and p24 were detected with the respective antibodies: PAI-7218 (Thermo Scientific, Erembodegem, Belgium), C8 and AB9044 (Abcam, Antwerp, Belgium). The monoclonal antibody directed to gp41, C8, was derived from HIV-1 gp41 Hybridoma (Chessie 8) cells and obtained through the NIH AIDS Reagent Program, Division of AIDS, NIAID, NIH, from Dr George Lewis [21]. Visualization was achieved by using secondary, horse radish peroxidase (HRP)-labelled antibodies, the SuperSignal West Pico Chemiluminescent Substrate kit (Thermo Scientific, Erembodegem, Belgium) and Image Lab (BioRad).

### Evaluation of the infectivity of the mutant virus strains

10^6^ C8166 cells were brought into wells of a 24-well plate at day 0 and infected with 40 ng p24 of wild-type (WT) or mutant HIV-1_NL4.3_. By adding RPMI-1640 culture medium it was ascertained that every well contained a total volume of 2 ml. Every day for 7 following days, cell culture samples were harvested and fixed in 3% formaldehyde. At the end, the percentage of infected cells was quantified for all samples by the analysis of eGFP expression upon infection with a FACS CantoII flow cytometer (Becton Dickinson, San Jose, CA). The data were analysed by FACS Diva Software (Becton Dickinson).

### Capture of HIV-1_NL4.3_ particles by Raji/DC-SIGN cells

Wild-type and mutant HIV-1_NL4.3_ strains (equivalents of 25 ng p24) were incubated with 10^6^ Raji/DC-SIGN cells for 1h at 37°C. Afterwards, the cells were washed thoroughly to eliminate unbound virions. The cells were then resuspended in 1ml of fresh culture medium, after which the concentration of the viral capsid protein p24 was quantified using a p24 enzyme-linked immunosorbent assay (ELISA) (PerkinElmer, Boston, MA), to quantify the amount of virus particles captured by the Raji/DC-SIGN cells. The virus capture efficiency was defined as the amount of DC-SIGN-captured virions per 10 ng p24 used during virus exposure to the cells, and expressed relative to WT virus.

### Transmission of captured virus from Raji/DC-SIGN to CD4^+^ T lymphocyte C8166 cells

Raji/DC-SIGN cells (2 x 10^5^) were exposed to WT or mutant HIV-1_NL4.3_ particles (equivalents of 5 ng p24) during 1h at 37°C. After thoroughly washing to eliminate unbound virions, the (virus-bound) cells were resuspended in 200μl of fresh culture medium. Next, the virus-bound Raji/DC-SIGN cells were exposed to 2 x 10^5^ C8166 cells in a total volume of 1 ml. Following an incubation of 72h, samples were harvested to quantify the amount of HIV-1 p24 antigen using a p24 ELISA (PerkinElmer). These values were used as a measure of viral production resulting from transmission of virions from virus-captured Raji/DC-SIGN cells to CD4^+^ C8166 cells.

### Statistics

A Student’s t-test was used to validate the significance of the data. A *p* value <0.05 was considered as significant.

## Results

### Several highly conserved *N*-linked glycans of gp120 are preferentially localized near disulphide bridges

Out of the 9 highly conserved disulphide bridges in gp120 of HIV-1_NL4.3_, four were found to contain at least 1 *N*-linked glycan on an asparagine directly neighboring one of the involved cysteines and at one of those four locations, both cysteines of the disulphide bridge were located directly next to a glycosylated asparagine. Besides these four cysteine bridges, one other disulphide bridge was found to be located near 2 glycosylated asparagines that were separated from the cysteines by only one other amino acid (Fig 1).

**Fig 2:**
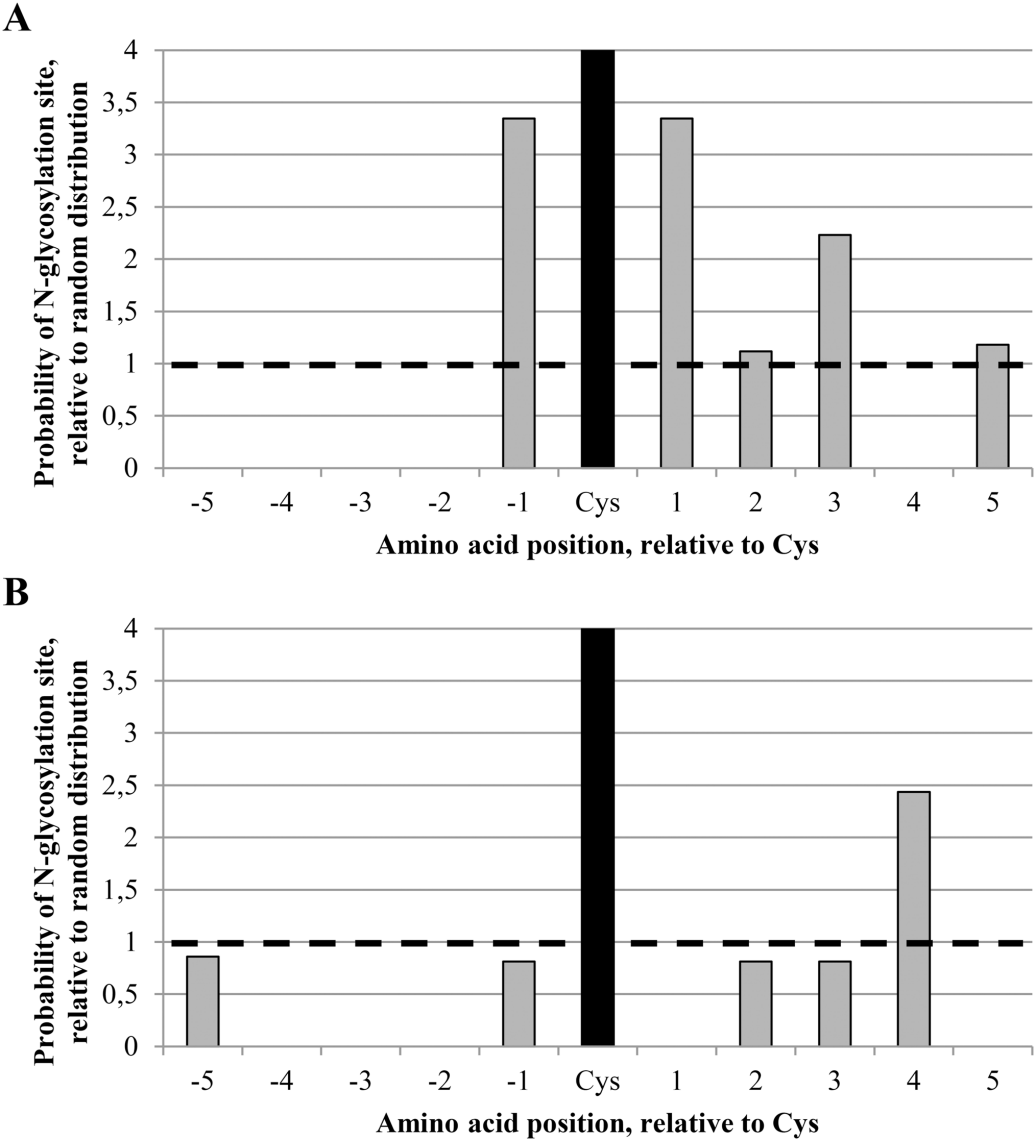
Probability of finding an *N*-linked glycosylation site at a position 1–5 amino acids from a disulphide bridge in gp120. The positions and levels of conservation of *N*-linked glycosylation sites in HIV-1 gp120 were determined using the N-glycosite software available on the HIV sequence database website [11], based on an alignment of consensus sequences of 19 HIV-1 subtypes (2004) [11]. The probability of finding *N*-glycosylation sites closeby cysteines involved in disulphide bridges was calculated. The graph shows the relative probabilities of a glycosylated asparagine at 1, 2, 3, 4 or 5 amino acid positions away from the cysteines involved in disulphide bridges. Negative amino acid positions correspond to positions at the N-terminal site of the cysteine, positive amino acid positions correspond to positions at the C-terminal site of the cysteine. The cysteine itself is shown as a black bar. The striped line indicates the probablities in case of random distribution of *N*-glycosylation sites. (A) *N*-glycosylation sites with at least 50% conservation. (B) *N*-glycosylation sites with less than 50% conservation.

An alignment of HIV-1 Env amino acid sequences was used to check the occurrence of neighboring disulphide bridges and *N*-glycans across gp120 belonging to a wide variety of HIV-1 strains. The alignment was obtained from the HIV sequence database [11], and the *N*-glycosylation sites in HIV-1 gp120 among 19 HIV-1 subtypes (including more than 200 individual strains) were determined. A distinction was made between *N*-glycosylation sites with at least 50% conservation and *N*-glycosylation sites with less than 50% conservation. A total of 24 conserved and 33 non-conserved *N*-glycosylation sites were found in HIV-1 gp120. Next, the probability of finding a conserved or non-conserved *N*-glycan at a distance of 1, 2, 3, 4 or 5 amino acids away from a disulphide bridge was calculated in case of random distribution of the *N*-glycans across gp120 (probability 1.0 at the ordinate of the distribution diagram, represented by the dashed horizontal line) (Fig 2A and 2B). These data were compared to the actual observed frequencies. It was found that the chance of finding a conserved *N*-glycan at an asparagine directly neighboring a cysteine which is involved in a disulphide bridge in gp120 (both at the N-terminal and the C-terminal side of the cysteine) was about 3.3 times greater than in the case of random distribution of the conserved *N*-glycans across gp120 (Fig 2A). At the N-terminal side, it was striking to notice that there are no conserved glycans at a distance of -2, -3, -4 or -5 amino acid positions from a disulphide bridge. The complete absence of an *N*-glycan at position -2 is expected because this is inherent to the obligate N-X-S/T *N*-glycosylation motif excluding an *N*-glycan 2 amino acids upstream of a cysteine. In contrast, the absence of conserved *N*-glycans at positions -3, -4 and -5 was not obvious and hence unexpected (Fig 2A). At the C-terminal side, there is a preference for *N*-glycosylation, especially at positions +1 and +3 (frequency 3.3 to 2.2 times higher than in case of random distribution). It could therefore be concluded that several conserved *N*-glycans of gp120 show a predominant localization near disulphide bridges, especially at the -1 amino acid position on the N-terminal side and the +1 and +3 amino acid positions at the C-terminal side of the cysteine, and that the complete absence of a conserved *N*-glycan at amino acid positions -3, -4, -5 and +4 was rather unexpected. Moreover, similar calculations were also performed for the non-conserved *N*-glycans of gp120. It was found that non-conserved glycans showed a less pronounced co-localization with disulphide bridges, except for the +4 amino acid position with respect of the involved cysteines (frequency at +4 position 2.4 times higher than in case of a random distribution) (Fig 2B). Although non-conserved glycans were observed at the -5 position with respect of disulphide bond-engaged cysteines, again no glycans were found at the -4 and -3 amino acid positions (Fig 2B).

For comparative reasons, similar calculations were also performed for other (highly *N*-glycosylated) envelope glycoproteins such as those of Ebola virus and HCV. For Ebola GP, not a single *N*-glycosylation site was found within a distance of 5 amino acids from a cysteine that was involved in a disulphide bridge, neither on the N-terminal or on the C-terminal side of the cysteines. Instead, for both envelope glycoproteins of HCV (E1 and E2) we found multiple *N*-glycans in proximity of disulphide bridges (S1 Fig). Although the co-localization of disulphide bridges and *N*-glycans was only modest for HCV E2, the phenomenon was more pronounced for HCV E1. In addition, for both E1 and E2 we observed more *N*-glycans at the first five positions near the C-terminal side of disulphide bridges, as compared to the N-terminal side. Thus, the pattern of *N*-glycan clustering near disulphide bridges in viral glycoproteins is not limited to HIV-1 gp120 on the one hand, but is not a general phenomenon either among viral glycoproteins.

### Site-directed mutagenesis of cysteines and neighboring *N*-glycosylation sites in HIV-1 gp120

In an attempt to reveal the function of disulphide bridges and the nearby *N*-glycans, site-directed mutagenesis on HIV-1_NL4.3_ gp120 was performed to mutate cysteines involved in these disulphide bridges, and to insert or eliminate *N*-glycosylation sites within a distance of 1 to 5 amino acids away from these cysteines (Fig 1 and S4 Table). For the elimination of disulphide bridges, one of the involved cysteines was mutated into an alanine. Deletion of *N*-glycans was achieved by mutating the asparagine which is part of the *N*-glycosylation motif into a glutamine. The insertion of new *N*-glycosylation sites was obtained by the introduction of an asparagine, 2 amino acids upstream of a newly introduced serine.

Fig 1 shows the location of the different mutations in HIV-1_NL4.3_ gp120. In total, 5 cysteines (red circles) and 7 asparagines (black arrows) were mutated, as well as combinations of both the cysteine and its neighboring asparagine(s). In addition, 6 new *N*-glycosylation sites (blue arrows) were introduced in the proximity of 2 disulphide bridges, either instead of, or in combination with, the *N*-glycosylation site that neighbors these cysteines in WT gp120.

**Fig 3:**
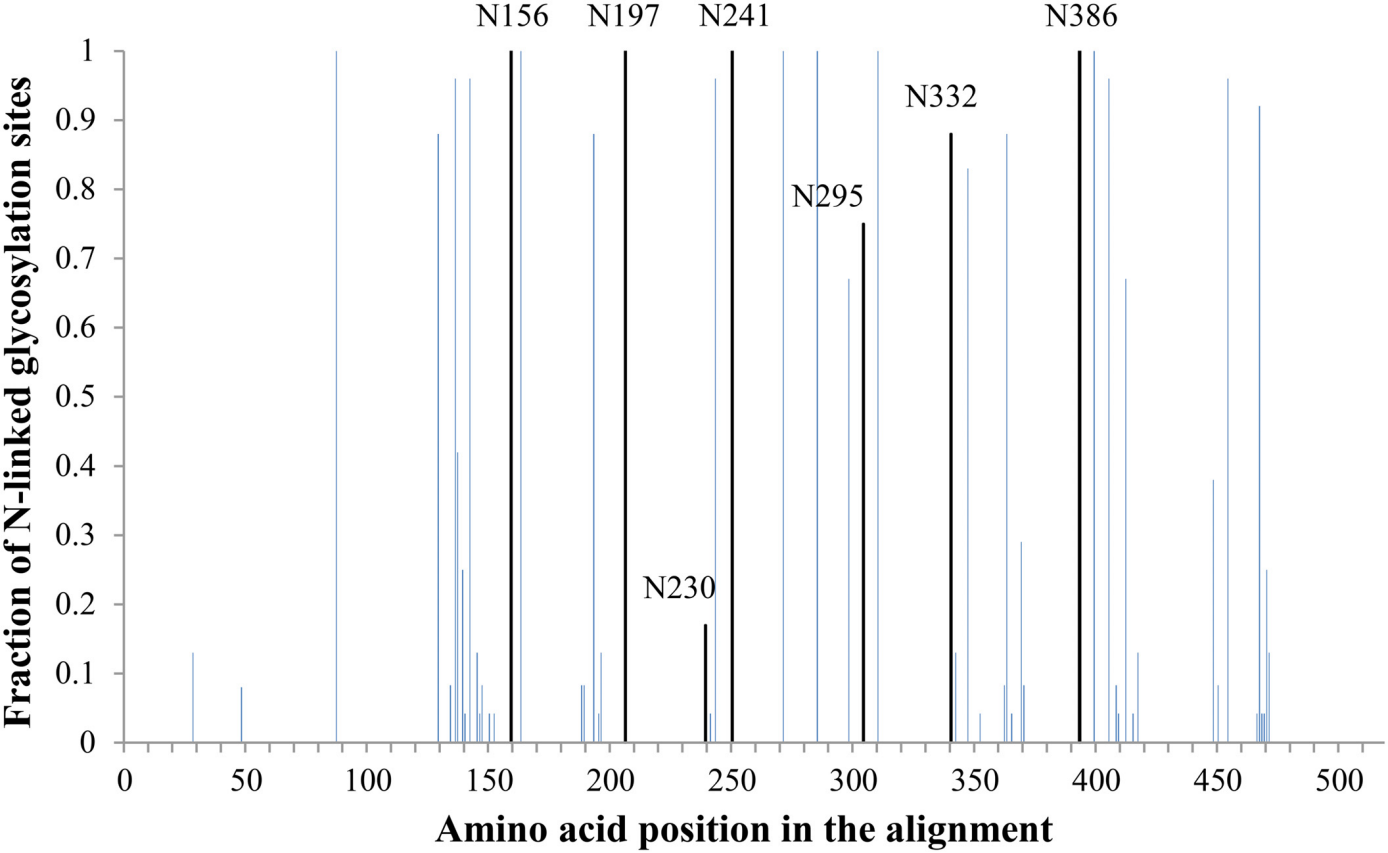
HIV-1 gp120 amino acid alignment with indication of *N*-glycosylation sites that were mutated in this study. An alignment of consensus sequences (2004) of the amino acid sequence of gp120 was obtained through the HIV sequence database [11] and was analyzed with the N-glycosite software [11] to locate *N*-glycosylation sites. For our analysis, we did not include the consensus of consensus sequences nor the ancestral sequences, but focused on the consensus sequences for HIV-1 subtypes A1, A2, B, C, D, F1, F2, G, H, CRF01-AE, CRF02-AG, CRF03-AB, CRF04-cpx, CRF06-cpx, CRF08-BC, CRF10-CD, CRF11-cpx, CRF12-BF, and CRF14-BG. The height of the bars indicates the level of conservation among these HIV-1 subtypes. The 7 black bars represent *N*-glycosylation sites that were found in HIV-1_NL4.3_ gp120 to be located near a disulphide bridge (directly neighbouring one of the disulphide cysteines or separated by only one amino acid) and were mutated by site-directed mutagenesis.

Fig 3 represents the alignment of HIV-1 gp120, obtained from the HIV sequence database [11], with indication of the *N*-glycans that were deleted using site-directed mutagenesis (vertical black lines with amino acid numbering). It was shown that 6 out of the 7 deleted *N*-glycans were at least 75% conserved [4 were fully (100%) conserved (i.e. N156, N197, N241 and N386), one was 88% conserved (i.e. N332) and the other *N*-glycosylation site was 75% conserved (i.e. N295)]. In contrast, the glycan on N230 which is present in HIV-1_NL4.3_ and included in our study, was only 17% conserved among all strains in the HIV sequence database [11].

### Effect of disulphide bridge or *N*-glycan deletions on the envelope glycoprotein levels in mutant viral particles

To investigate the role of the co-localization of disulphide bridges and *N*-glycans in gp120 of HIV-1_NL4.3_, WT virus was compared to mutant viruses in which either the disulphide bridge, the nearby *N*-glycosylation site or both were mutated. First, the effect of these mutations on envelope glycoprotein biosynthesis and subsequent incorporation in the envelope of the viral particles was examined using western blotting on lysates of WT and mutant virus strains.

**Fig 4:**
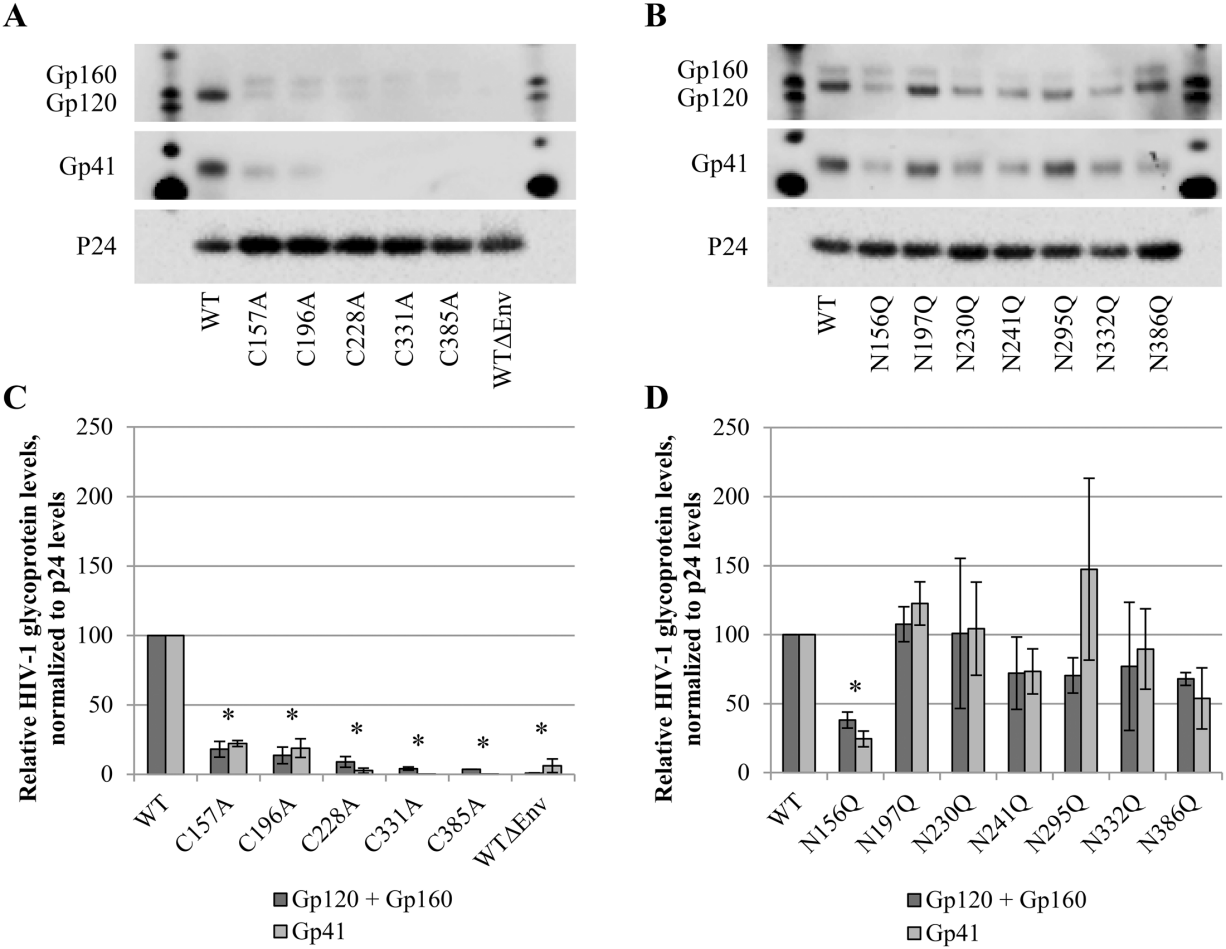
Western blot analysis of envelope glycoprotein incorporation in WT and mutant virus particles. Virus was concentrated, lysed and subjected to western blotting. Gp120, gp41 and p24 were detected in virus lysates of (A) mutants lacking a disulphide bridge and (B) *N*-glycosylation site mutants. (C-D) Quantification of protein levels was performed using the BioRad Image Lab Software, based on panels A and B. Gp160, gp120 and gp41 levels are presented after normalization to the p24 levels. Graphs represent the mean ± SEM based on 2–4 independent experiments. The difference between WT and mutant virus was considered to be significant when the *p* value calculated using the student’s t-test was <0.05 (* = p<0.05, for both gp120+gp160 and gp41).

It was shown that the disruption of the disulphide bridges by mutating one of its cysteines markedly abrogated (by 75 to >95%) incorporation of both gp120 and gp41 in the virus particles (Fig 4A and 4C). In contrast, most of the *N*-glycan deletions did not markedly reduce envelope glycoprotein expression and incorporation in the viral particle (Fig 4B and 4D). Only the mutation N156Q in gp120 resulted in a substantial decrease of gp120 (by ∼ 60%) and gp41 (by ∼ 75%) levels in the viral envelope.

It could be concluded that deletion of a disulphide bridges had a much more dramatic influence on the eventual incorporation of gp120 and gp41 in the virus particles, as compared to the deletion of the neighboring *N*-glycans.

### Disulphide bridges or *N*-glycan deletions have a variable effect on viral infectivity

**Fig 5:**
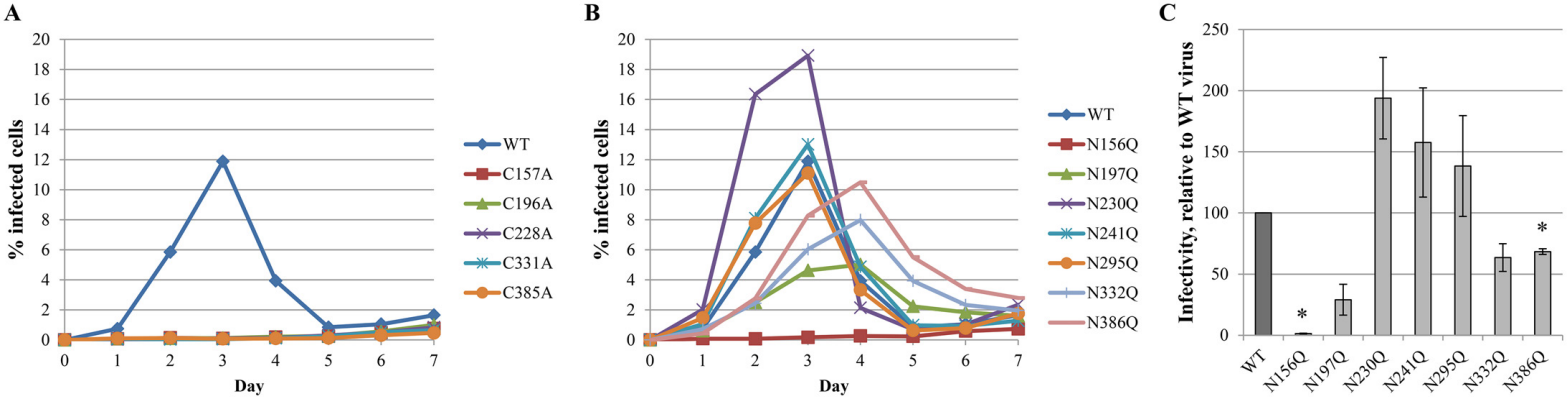
Infectivity of WT and mutant gp120 HIV-1 strains. At day 0, CD4^+^ T lymphocyte C8166 cells were infected with similar viral loads of WT or mutant virus strains, based on equal amounts of the p24 capsid protein. For 7 consecutive days, samples were harvested, fixed and subjected to flow cytometry to analyse eGFP expression. (A) Infectivity of mutant viruses lacking disulphide bridges in gp120, due to the mutation of one of the involved cysteines into an alanine. (B) Infectivity of mutant viruses lacking one *N*-glycosylation site in gp120 due to the mutation of the asparagine of the glycosylation motif into a glutamine. (C) The infectivity as shown in panel B was quantified using a linear regression to part of the curves, from day 1 post infection to the peak of the infectivity curve. Data are the means ± SEM of at least 2 independent experiments. The difference between WT and mutant virus was considered to be significant when the *p* value calculated using the student’s t-test was <0.05 (* = p<0.05).

Next, the influence of the deletion of disulphide bridges and/or their neighboring *N*-glycans was determined on viral infectivity. Therefore, WT and mutant HIV-1_NL4.3_ were exposed to susceptible CD4^+^ T cells for an extended time period. Each day, part of the cell cultures were harvested and viral infection was quantified based on eGFP expression in the infected cells. As could be predicted from the western blot analysis of the gp120/gp41 levels in the mutant viruses, those mutant virus strains lacking one of the disulphide bridges in gp120 virtually completely lost their infectivity potential (Fig 5A). The effect of the deleted neighboring *N*-glycans on virus infectivity was dependent on the particular location of the deleted *N*-glycans (Fig 5B and 5C). For example, the gp120 mutations N197Q and in particular N156Q resulted in a markedly decreased viral infectivity, relative to WT virus. Gp120 mutations N332Q and N386Q also showed decreased viral infectivity, although less pronounced. Instead, the gp120 mutations N230Q, N241Q and N295Q were shown to have an enhancing effect on viral infection, as compared to WT virus.

### Deletion of a disulphide bridge, but not a nearby *N*-glycan, markedly affects virus capture efficiency by DC-SIGN

Not only infectivity as such, but also the capacity of HIV to be efficiently captured by DC-SIGN and its subsequent transmission to susceptible cells is important during *in vivo* viral infection. DC-SIGN is a lectin of the innate immune system that is present on dendritic cells and recognizes high-mannose-type glycans on pathogen targets such as HIV gp120 [16, 22]. The deletion of a high-mannose-type glycan on gp120 can potentially influence the capability of DC-SIGN^+^ cells to capture mutant virus particles. To explore this hypothesis, WT and mutant virus particles were exposed to Raji/DC-SIGN cells during one hour, after which the cells were washed thoroughly to eliminate unbound virions. The amount of captured virus was then quantified using a p24 ELISA. It was shown that the deletion of a disulphide bridge resulted in a marked decrease in the capture efficiency (30–81% decrease), as compared to WT virus (Table 1, left part). Instead, Table 1 (right part) reveals that the deletion of an *N*-linked glycan located in very close proximity of a disulphide bridge in most cases did not markedly reduce the capture efficiency by DC-SIGN, as compared to WT virus. Interestingly, the mutations N197Q and N295Q in gp120 even increased the capacity of the mutant viruses to become captured by Raji/DC-SIGN cells.

**Table 1:** Capture efficiency of mutant gp120 virus strains, relative to capture efficiency of wild-type virus.

Virus	Relative capture efficiency	Virus	Relative capture efficiency
C157A	59 ± 3 (*)	N156Q	106 ± 7
C196A	70 ± 4 (*)	N197Q	167 ± 16
C228A	19 ± 2 (*)	N230Q	108 ± 12
C331A	30 ± 7 (*)	N241Q	100 ± 7
C385A	46 ± 12	N295Q	130 ± 16
		N332Q	91 ± 8
		N386Q	93 ± 11

Data are the means ± SEM of at least 2 independent experiments.

The difference between WT and mutant virus was considered to be significant when the *p* value calculated using the student’s t-test was <0.05 (* = p<0.05).

### Effect of disulphide bridge or *N*-glycan deletions on transmission of DC-SIGN-captured virus particles

**Fig 6:**
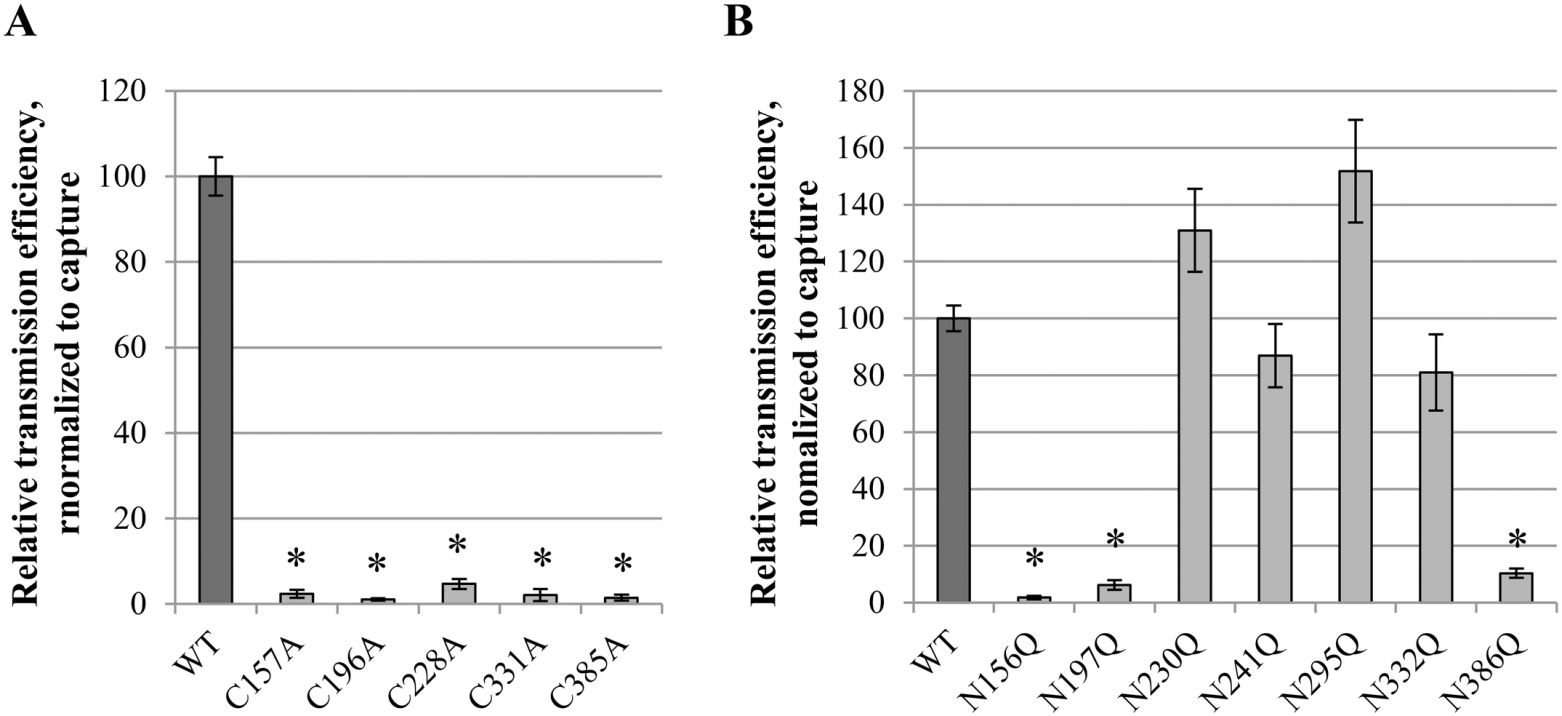
Transmission efficiency of wild-type and mutant virus strains. Virus-exposed Raji/DC-SIGN cells were cocultivated with C8166 CD4^+^ T cells for 72 h. Production of virus particles by the C8166 cells (following transmission of HIV from the virus-captured Raji/DC-SIGN cells) was quantified using a p24 ELISA and was used as a measurement for transmission efficiency. Data were normalized to capture efficiency and presented as relative to transmission of WT virus. Data are the means ± SEM of at least 2 independent experiments. The difference between WT and mutant virus was considered to be significant when the *p* value calculated using the student’s t-test was <0.05 (* = p<0.05).

When virus-captured Raji/DC-SIGN cells are brought into contact with CD4^+^ T lymphocyte cells, virus is efficiently transmitted from the Raji/DC-SIGN cells to the susceptible CD4^+^ T cells, which are subsequently infected and produce new viral particles [16]. The efficiency of viral transmission upon cocultivation of virus-captured Raji/DC-SIGN cells and CD4^+^ C8166 cells can therefore be quantified by analyzing virion production by the C8166 cells through a p24 ELISA. The transmission efficiency was normalized to initial equal capture efficiencies (as determined in the previous section). It was found that the deletion of a disulphide bridge in HIV-1 gp120 almost completely prevented the viral transmission to uninfected CD4^+^ cells (Fig 6A). Interestingly, the glycosylation site mutations N156Q, N197Q and N386Q in gp120 had a dramatic compromising effect on the mutant virus transmission efficiency, as compared to WT virus. Gp120 mutations N241Q and N332Q were endowed with transmission efficiencies comparable to WT virus. In contrast, gp120 mutations N230Q and N295Q resulted in increased virus transmission (Fig 6B).

### Correlation between viral infectivity and transmission efficiency for mutant viruses with *N*-glycan deletions in gp120

**Fig 7:**
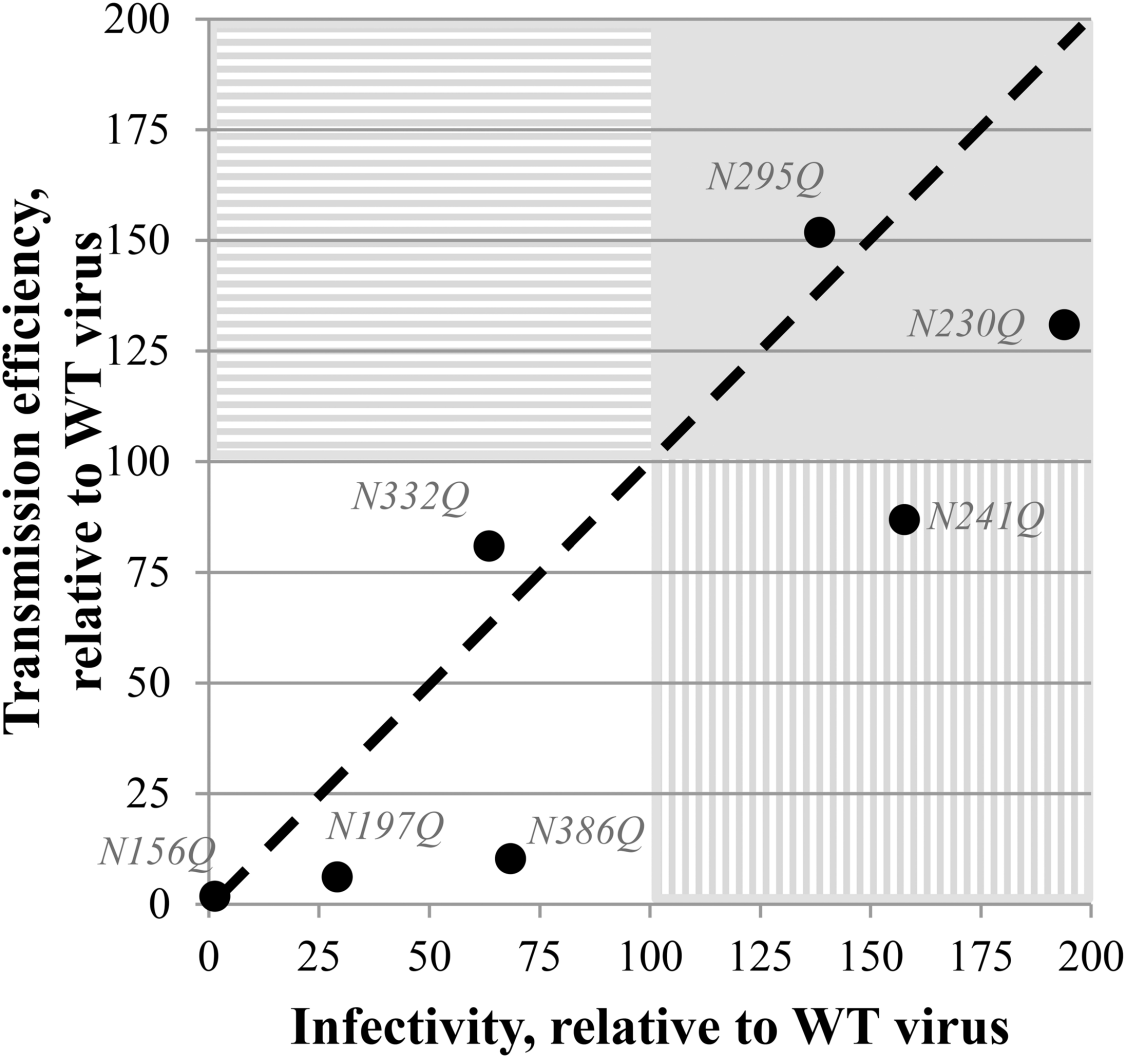
Correlation between the infectivity and transmission efficiency of the gp120 *N*-glycan deleted mutant virus strains, relative to WT virus. The black dashed line represents a curve where infectivity and transmission would have been equally influenced by the mutations. White quadrant: both transmission and infectivity are decreased. Full grey quadrant: both transmission and infectivity are increased. Quadrant with horizontal striping: transmission efficiency is increased while infectivity is decreased. Quadrant with vertical striping: infectivity is increased while transmission efficiency is decreased.

It could be assumed that there would be a close correlation between the effect of gp120 *N*-glycan deletions on viral infectivity and transmission efficiency, since both processes involve the interaction between gp120 and CD4/CXCR4/CCR5, require conformational changes in gp120 upon interaction with CD4/CXCR4/CCR5 and result in membrane fusion. Whereas the majority of the *N*-glycan-deleted virus mutants showed a decreased infectivity and a decreased transmission efficiency (N156Q, N197Q, N332Q, N386Q), two virus mutants (N230Q and N295Q) were endowed with both an increased infectivity and transmission potential (Fig 7). Interestingly, it looks like most HIV-1 mutants (i.e. N197, N230Q, N241Q and N386) have consistently a more pronounced deleterious effect on transmission efficiency than on infection efficiency. This difference in efficiency was most pronounced for the mutant gp120 N386Q HIV-1 strain that showed an infection potential ~70% of WT virus, while its transmission potential was only 10% of WT virus (Fig 7).

### The effect of newly introduced gp120 *N*-glycans in proximity of disulphide bridges on viral function

In Fig 2A, we demonstrated that conserved *N*-glycans can be preferentially found in close proximity of disulphide bridges in gp120, although some positions (mainly N-terminal of the cysteines: -5, -4 and -3) seem to be excluded for *N*-glycosylation. When taking into account non-conserved *N*-glycans, *N*-glycans were still absent at positions -4 and -3 with respect of disulphide bond-engaged cysteines of gp120 (Fig 2B). To investigate the meaning of the consistent lack of *N*-glycans at specific positions in gp120 near disulphide bridges, mutant HIV-1_NL4.3_ strains were generated in which an *N*-glycosylation site was inserted in gp120 at those positions. Thus, *N*-glycans were inserted at positions -5, -4 and -3, near one of the cysteines involved in a disulphide bridge (Fig 1 and S4 Table). Next, the influence of these introduced *N*-glycans in HIV-1 gp120 on viral infectivity was studied.

**Fig 8:**
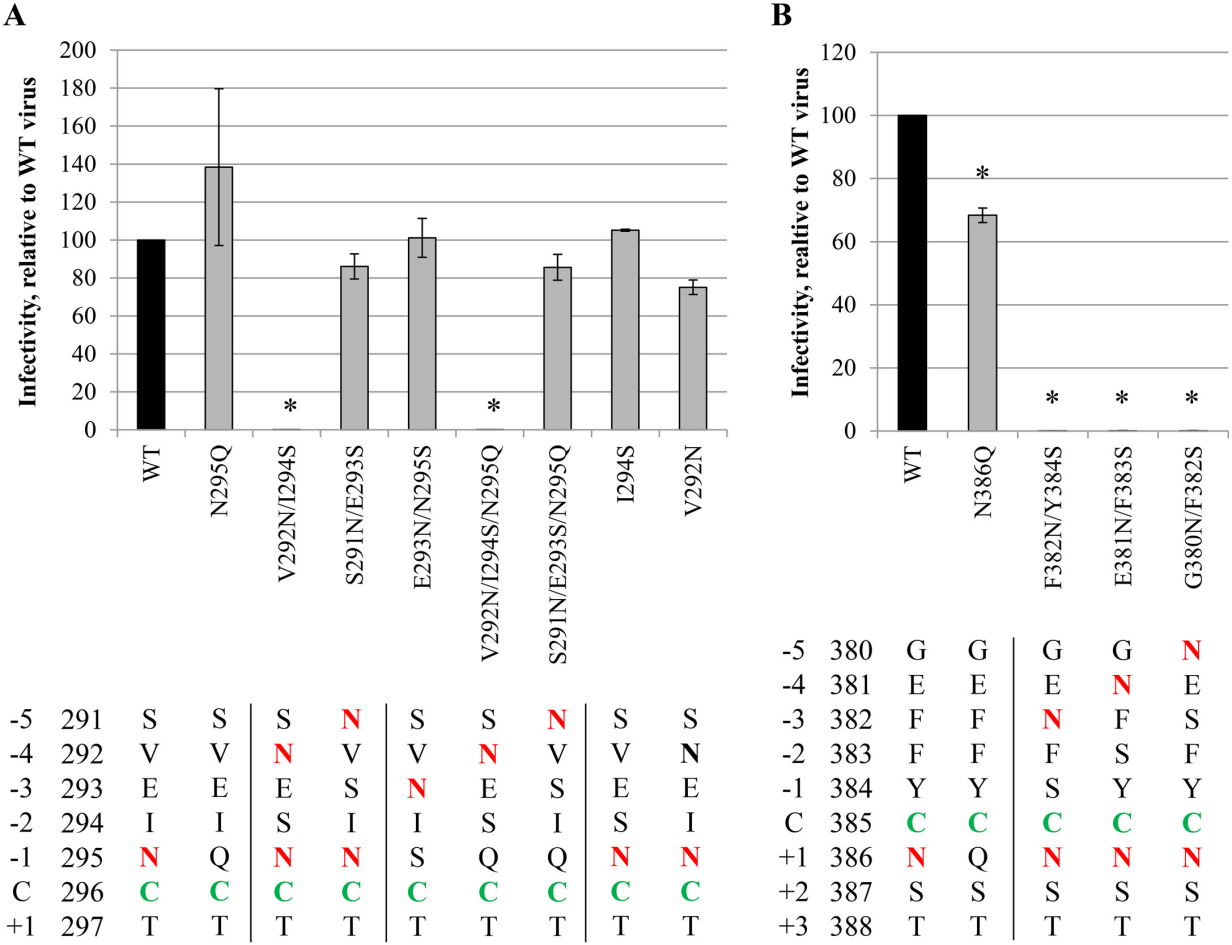
Infectivity of mutant HIV-1_NL4.3_ strains with *N*-glycosylation sites introduced in gp120 close to, and upstream of, the disulphide-involved C296 (A) and C385 (B). At day 0, CD4^+^ T lymphocyte C8166 cells were infected with similar viral loads of WT or mutant virus strains, based on equal amounts of the p24 capsid protein. For 7 consecutive days, samples were harvested, fixed and subjected to flow cytometry to analyse eGFP expression. The infectivity was quantified using a linear regression to part of the obtained eGFP-expression curves, from day 1 post infection to the peak of the curve. Asparagines involved in *N*-glycosylation sites (N-X-S/T) are coloured red. C296 and C385 are indicated in green. One introduced Asn (V290N) that was not part of an *N*-glycosylation site is indicated in bold **(A)**. Data are the mean ± SEM of 2–3 independent experiments. The difference between WT and mutant virus was considered to be significant when the *p* value calculated using the student’s t-test was <0.05 (* = p<0.05).

Fig 8A shows the effect on viral infectivity of *N*-glycans that were introduced at the N-terminal side of cysteine 296 that is involved in a disulphide bridge with cysteine 331. In WT gp120, C296 is flanked by an *N*-glycan at N295. *N*-glycosylation sites were introduced in WT gp120 and in gp120 lacking the N295 glycan. As was shown before (Fig 5B and 5C), the deletion of the N295 glycan did not have a detrimental effect on viral infectivity. The introduction of an *N*-glycan at position -5 had no effect on viral infectivity, irrespective of the absence or presence of the N295 glycan. However, the introduction of an *N*-glycan at position -4 resulted in a nearly complete abolishment of viral infectivity. This was found upon the introduction of the glycosylation site in both WT gp120 and in gp120 lacking the N295 glycan. To make sure that the observed effect on viral infectivity was due to the introduction of an *N*-glycan and not to the individual mutations V292N or I294S, both mutations were also introduced separately into WT gp120. It was found that the sole mutations V292N or I294S had no effect on viral infectivity. Therefore, we conclude that the loss of viral infectivity of the mutant HIV-1 strains V292N/I294S and V292N/I294S/N295Q was due to the insertion of a functional *N*-glycosylation site at a position in gp120 that is normally highly disfavored for *N*-glycosylation. An *N*-glycan was also introduced at position -3, thereby inevitable deleting the N295 glycan (N295S mutation). This newly introduced glycosylation site had no effect on viral infectivity.

One other disulphide bridge in gp120 was selected for the introduction of nearby *N*-glycosylation sites, namely the C385-C418 disulphide bridge. New *N*-glycosylation sites were generated at the N-terminal side of cysteine 385, at amino acid positions -5, -4 and -3. It was found that these 3 *N*-glycosylation sites all resulted in the complete loss of viral infectivity of the mutant virus strains (Fig 8B).

Together, these data indicate that the absence of *N*-glycans at positions close to, but upstream of, disulphide bridges in gp120 is not a simple coincidence since the introduction of *N*-glycans at these positions in most cases resulted in the loss of viral infectivity.

## Discussion

We observed for HIV-1_NL4.3_ that many of the disulphide bridges in gp120 co-localize with *N*-linked glycans. We confirmed this finding using a large alignment, containing Env amino acid sequences of ~200 HIV-1 strains belonging to 19 different HIV-1 subtypes [11]. We found a statistically significant predominance of conserved *N*-linked glycans directly neighboring a disulphide bridge, or in close proximity at the C-terminal side of at least one of its cysteines. Moreover, no conserved glycans were found in gp120 at positions -5, -4, -3, -2 or +4, in respect to cysteines involved in a disulphide bridge. Although this could be expected for position -2 due to the nature of the *N*-glycosylation motif (Asn-X-Ser/Thr, in which X is any amino acid except a proline and thus, a cysteine at position 0 prevents the appearance of a glycan at position -2), the lack of conserved glycans at positions -5, -4, -3 and +4 was rather unexpected. However, we found non-conserved *N*-linked glycans at positions -5 and +4 neighboring disulphide bridges in gp120, indicating that occasionally, non-conserved glycans may occur at these positions. For HCV E1, we found a similar predominant correlation between the location of disulphide bridges and nearby *N*-glycans as for HIV-1 gp120, a phenomenon being less pronounced for HCV E2. In contrast, we found no co-localization of disulphide bridges and *N*-glycans at all in the envelope glycoprotein of Ebola virus.

In addition to the probability calculations that were performed for HIV-1 gp120, HCV E1 and E2, and Ebola virus GP, we aimed to perform a similar calculation for glycosylated Influenza A virus heamagglutinin (HA). However, based on Daniels *et al*. [23], and confirmed by various HA sequences obtained from the NCBI database [12], the number of *N*-glycans on Influenza A virus HA was too limited, as compared to the number of disulphide bridges, to obtain relevant data on the probability of co-localization of disulphide bridges and *N*-glycans in HA. We could conclude that some viral glycoproteins such as HIV-1 gp120 and HCV E1 have a strong preference of encoding an *N*-glycan near some of its disulphide bridges, a phenomenon that might have a particular reason in those cases (see below).

It should be noticed that the conserved *N*-glycans that were shown to co-localize with disulphide bridges in HIV-1 gp120 are not necessarily 100% conserved. This implies that some HIV-1 strains or subtypes do not strictly depend on the presence of these *N*-glycans. However, when inspecting the positions and levels of conservation of *N*-glycans across the 19 investigated HIV-1 subtypes (including recombinants), it is striking that the lack of a particular conserved *N*-glycan in strains of one HIV-1 subtype is often associated with the presence of a non-conserved *N*-glycan nearby the missing conserved gp120 *N*-glycan in these strains. One interesting example of this observation is the glycan at asparagine 340 in the consensus of consensus HIV Env sequence [11] (position 332 in HIV-1_HXB2_), which is positioned at the +1 position with respect of the disulphide bond-engaged cysteine 339. This *N*-glycan is 84% conserved among the 19 HIV-1 subtypes included in our alignment, but is absent in subtypes A2, F2 and CRF01-AE. Interestingly, in these HIV-1 subtypes, a non-conserved glycan is found at the asparagine 342 position, which is only 2 amino acid positions downstream of asparagine 340. This suggests that the incomplete conservation of *N*-glycans of gp120 can be compensated by non-conserved *N*-glycans at nearby positions.

Using site-directed mutagenesis, we generated HIV-1_NL4.3_ mutants either lacking disulphide bridges and neighboring *N*-glycans in gp120, or carrying an additional *N*-glycan at a position in gp120 that is normally disfavored for *N*-glycosylation. In total, 5 disulphide bridges and 7 *N*-glycosylation sites were mutated, and 6 *N*-glycosylation sites were newly generated. The effect of these mutations was then studied using an array of assays evaluating viral function. It was shown that the deletion of the 5 disulphide bridges that were found in close proximity to one or more *N*-glycans ultimately led to a severe decrease in all examined viral functions: envelope glycoprotein expression, viral infectivity, capture of virus particles by DC-SIGN and transmission of DC-SIGN-captured virus to T cells. These data are in agreement with the data of Van Anken *et al*. [6] who showed that 5 out of the 9 gp120 disulphide bridges were indispensable for gp120 folding and another 2 disulphide bridges were crucial for the correct function of the produced gp120. Interestingly, these investigators showed that the deletion of the gp120 C385-C418 disulphide bridge resulted in severe folding deficiencies, although this mutant virus strain was shown to still have some residual viral infectivity. The 5 disulphide bridges that were included in our study were all described by Van Anken *et al*. [6] as being indispensable (or in case of C385-C418 at least to some extent) for the functionality of gp120. Three of these disulphide bridges (i.e. C228-C239, C296-C331 and C385-C418) were also found to be crucial for the correct folding and biosynthesis of gp120. In contrast, disulphide bridges C126-C196 and C131-C157 were reported not to be crucial for gp120 folding. Comparing these data with the western blot data obtained in our studies, we could partially confirm the findings of Van Anken *et al*. [6]. We also found a more pronounced defect on envelope glycoprotein biosynthesis for the disulphide bridges C228-C239, C296-C331 and C385-C418. However, also upon the disruption of the disulphide bridges C126-C196 and C131-C157 we found substantially decreased glycoprotein levels in the viral envelope of the mutant virus particles, suggesting that the lack of these disulphide bridges also resulted in a heavily compromised gp120/gp41 biosynthesis or envelope incorporation into the virus particles. Van Anken *et al*. [6] investigated gp160/gp120 biosynthesis using a vaccinia-virus-based expression system, encoding the viral *Env* gene. They studied the folding of intracellular pools of gp160/gp120 and the shedding of gp160/gp120 in the cellular supernatants. However, the incorporation of gp160/gp120/gp41 in viral particles was not studied before. This suggests that possibly, the deletion of the disulphide bridges C126-C196 and C131-C157 might indeed enable correct folding (as observed by Van Anken *et al*. [6]) but could result in a deficient incorporation of the envelope glycoproteins in the viral envelope of newly produced virions (as observed in our studies). Furthermore, the role of the gp120 disulphide bridges has also been studied by others, emphasizing the crucial importance of most gp120 disulphide bridges for the biosynthesis of gp120 and the infectivity of the virus [24–25]. Our data confirm the indispensable role of the C126-C196, C131-C157, C228-C239 and C296-C331 disulphide bridges for the viral functions, as previously reported. However, we did not find significant residual viral replication for a mutant HIV strain that lacks the C385-C418 disulphide bridge, which was different from what has been earlier reported by others [6, 24, 26].

In order to generate HIV-1_NL4.3_ mutants with deleted disulphide bridges, we mutated one of the involved cysteines into an alanine, thereby disrupting the particular disulphide bridge. Theoretically, the loss of only one single cysteine could result in the scrambling of the disulphide bridges, resulting in a folded (or misfolded) mutant gp120 containing ether (non-native) disulphide bridges. However, Van Anken *et al*. have compared the effect of the single mutation of one of the disulphide bond-engaged cysteines with the effect of mutating both cysteines, and obtained highly similar gp120 folding kinetics for both the single and the double mutants [6]. They therefore suggested that non-native disulphide bridges are not readily formed upon the mutation of only one disulphide bond-engaged cysteine. If non-native disulphide bridges would occur at all, they were suggested to have a transient nature, breaking down easily during further oxidative folding of gp120 [6]. This was confirmed by other investigators, who demonstrated that the connectivity pattern between gp120 cysteines is highly conserved, except for the disulphide bridges in the V1-V2 region, which occur with variable connectivities [27–28].

HIV-1_NL4.3_ mutants lacking the *N*-glycosylation sites neighboring disulphide bridges were also generated. It was shown that the N156 glycan was the only investigated glycan that was indispensable for the correct expression/incorporation of the envelope glycoproteins and the N156, N197 and N386 glycans were found to play an important role for the proper function of the envelope glycoproteins. Our data largely confirm the results of Wang *et al*. [29]. They came to the same conclusions regarding the N156Q and N197Q mutations in HIV-1 gp120. However, they found a significant decreased envelope gp120 level in N241Q mutated virus, while we obtained viral gp120 levels that were similar to WT virus. In addition, Wu *et al*. demonstrated the requirement of the N156 glycan for the correct formation of the C131-C157 disulphide bridge during the biosynthesis of gp120 [30], a finding that is in full agreement with the highly compromised infectivity we observed for the N156Q gp120 mutated virus.

Despite the significantly decreased expression and incorporation of N156Q gp120 in the envelope of mutant viral particles, the DC-SIGN-mediated capture of the N156Q gp120 mutant virus strain had an efficiency highly comparable to WT virus. Since DC-SIGN-mediated virus capture is dependent on the interaction of DC-SIGN with gp120, these data might seem contradictory. However, a similar phenomenon was demonstrated before for the N616Q glycan deletion in gp41 [31]. This glycan deletion resulted in a >85% decrease in HIV envelope glycoprotein levels in the viral particle, while only moderately (20%) decreasing the DC-SIGN-mediated capture efficiency [31]. These data therefore suggest that (i) the interaction between DC-SIGN and gp120 is highly efficient, enabling a WT-like capture efficiency with only a minimal amount of gp120 available for interaction with DC-SIGN, and/or (ii) that the expression level of gp120 on WT virus particles exceeds the binding capacity of the limited number of DC-SIGN molecules expressed on dendritic cells.

An in-depth study of the role of the highly conserved N262 glycan of HIV gp120 has previously been performed, indicating that the deletion of this *N*-glycan results in a deficient cleavage of the mutant gp160 into gp120 and gp41 in the Golgi apparatus [32]. Indeed, structural data recently obtained by X-ray crystallography demonstrated the central role of the N262 glycan in the folding and stabilization of HIV-1 gp120 [33]. Furthermore, it was demonstrated that the N262-deleted gp160 was targeted for lysosomal degradation, which is in agreement with the apparent absence of N262-deleted gp160 or gp120 on the viral envelope [32]. It would therefore also be interesting to study in more detail the biosynthesis of the mutant HIV-1 gp160 or gp120 with a deleted N156 glycosylation site. The examination of the oxidative folding, signal peptide cleavage, glycosylation, dimerization and processing of mutant gp160 into gp120 and gp41 would provide a better understanding of the role of the N156 glycan during HIV gp160 or gp120 biosynthesis.

Interestingly, we found an increased infectivity and transmission potential for the HIV-1 gp120 mutants lacking the N230 or N295 glycans. It might not be a coincidence that these *N*-linked glycans have been reported to be readily deleted during the course of resistance development towards carbohydrate-binding agents (CBAs) [34]. These compounds have been shown to be potent HIV entry inhibitors by interacting with the glycans on HIV-1 gp120 [35]. CBA resistance development is associated with the deletion of multiple gp120 *N*-linked glycans, predominantly high-mannose-type glycans. As reported by Férir *et al*., the gp120 N230 and N295 glycans are amongst the most frequently deleted glycans during CBA resistance development [34]. The increased infectivity and transmission potential of HIV strains lacking the single N230 or N295 glycans of gp120, as found in our study, might therefore suggest that these mutations occurred as secondary mutations during CBA resistance development, compensating for the detrimental effects of the primary CBA resistance mutations on the viral infectivity, which has already been suggested for the N230 glycan by Hu *et al*. [36].

In addition to the mutants lacking either 1 *N*-glycan or 1 disulphide bridge, we also generated HIV-1_NL4.3_ mutants lacking the disulphides in combination with their neighboring *N*-glycans. We analyzed the infectivity of these virus strains and could conclude that the absence of the disulphide bridges, either in the presence or the absence of the neighboring *N*-glycan(s), was invariably highly detrimental for the infectivity of the mutant virus strains. The combined deletion of both the disulphide bridge and the neighboring *N*-glycan was therefore never able to rescue the defect caused by the deletion of the disulphide bridge (data not shown).

The functional properties observed for several *N*-glycans located close to the disulphide bridges is intriguing. It has earlier been shown that (co)receptor-induced conformational changes in gp120 during virus entry are mediated through the oxidoreduction of certain disulphide bridges of gp120 by cellular oxidoreductases such as protein disulphide isomerase (PDI), thioredoxin-1 (Trx1) and glutaredoxin-1 (Grx1) [37–40]. It has been suggested that the disulphide bridges C119-C205, C126-C196 and C385-C418 are preferentially targeted by these enzymes [41]. It is interesting to notice that C126-C196 and C385-C418 were both included in our study because of their close proximity to the *N*-glycosylation sites containing N197 and N386, respectively. It might not be a coincidence that these *N*-glycans were found to have a crucial role for at least some viral functions. Both glycan deletions (N197Q and N386Q) were indeed found to have detrimental effects on viral infectivity and in particular on transmission of DC-SIGN-captured mutant virus particles to CD4^+^ T cells. These effects could not simply be attributed to a defective envelope glycoprotein synthesis (>50% gp120/gp41 present in the mutant virus particle), as was the case for the N156Q mutation in gp120 (<50% gp120/gp41 present in the mutant virus particle).

In addition to the deletion of *N*-linked glycans neighboring disulphide bridges in gp120, we have also introduced new *N*-glycosylation sites in gp120, at positions close to disulphide bridges that are normally disfavored (excluded) for *N*-glycosylation. The six HIV-1_NL4.3_ mutants that were generated are characterized by the addition of an asparagine that could be glycosylated at position -5, -4 or -3 of a cysteine involved in a disulphide bridge in gp120. Two disulphide bridges were selected for the generation of these mutants: C296-C331 and C385-C418. *N*-glycosylation sites were either introduced upstream of C296 or upstream of C385. It was found that one of the three mutants with an additional *N*-glycan upstream of C296 lost its infectivity. This result was independent of the presence of the N295 glycan that is neighboring C296 in WT gp120. Strikingly, the three mutants in which a glycosylation site was introduced upstream of C385 were all found to lack residual infectivity. These findings indicate that the presence of *N*-glycans at specific unfavorable positions in gp120, near disulphide bridges, can be highly detrimental for the functionality of gp120. It might not be a coincidence that the deleterious effects of the introduced *N*-glycans were more pronounced when positioned upstream of C385. As noted above, this disulphide bridge is targeted by cellular oxidoreductases during the HIV entry process [41]. Deletion of the neighboring N386 glycan also had detrimental effects on viral infectivity. Therefore, it might be attractive to hypothesize that the presence or absence of glycans in close proximity to some disulphide bridges might have a crucial influence on the accessibility of the disulphide bridge by oxidoreductases. The presence of *N*-glycans at unfavorable positions, or the absence of *N*-glycans at favorable positions near disulphide bridges might therefore affect oxidoreduction-driven conformational changes that are required to allow efficient entry and transmission of the virus to susceptible CD4^+^ cells. Thus, in particular, we might hypothesize that the N197 and N386 glycans fulfill crucial functions during HIV infection/transmission by regulating the oxidoreduction of the neighboring disulphide bridges in gp120, which is a crucial step in the viral entry step. However, more research will be needed to validate this hypothesis.

A similar spatial organization of *N*-glycans and disulphide bridges was also found for the HCV envelope glycoprotein E1, and somewhat less pronounced for glycoprotein E2. Although these envelope glycoproteins have been shown to mediate viral entry in an oxidoreductase-independent manner, the oxidized *versus* reduced state of their disulphide bridges has been shown to be crucial for HCV entry [42]. Fraser *et al*. demonstrated that a minimum of 1 unbound cysteine has to be present in both E1 and E2 to allow efficient viral infection. They suggest that these free thiol-groups are obligate to enable acid-induced conformational changes in E1 and E2, upon receptor binding and subsequent endocytosis of the viral particle. Acidification of the endosome may cause free thiol-groups to evoke thiol-disulphide isomerizations, ultimately leading to the fusion of the viral and endosomal membranes. Therefore, the observed co-localization of disulphide bridges (or unbound cysteines) and *N*-glycans in HCV E1 (and E2) might be important for the regulation of disulphide bridge formation *versus* unbound cysteine residues as well. In contrast to HIV gp120 and HCV E1 and E2, no *N*-glycans were found in proximity to disulphide bridges in the Ebola glycoprotein GP. Thiol-disulphide exchange reactions have, to our knowledge, not been shown to be involved in the Ebola entry process. Upon endocytosis of Ebola virus particles, acidification induces cleavage of GP by the cellular proteases Cathepsin B and L [43]. However, this proteolysis on itself is not sufficient to mediate fusion of the viral and endosomal membranes. Therefore, it has been suggested that an additional host cell factor is needed to mediate the conformational changes in GP which allow membrane fusion [44]. Disulphide reduction has so far not been demonstrated for the GP of Ebola virus [45].

It would be interesting to examine the occurrence of *N*-linked glycans near disulphide bridges in the three-dimensional structure of fully glycosylated HIV-1 gp120. This structure has recently been published by Lyumkis *et al*. [46] and Kong *et al*. [33], and was generated using cryo-electron microscopy and X-ray crystallography, respectively. A careful 3D analysis of the spatial organization of disulphide bridges and *N*-glycans in HIV gp120 would contribute to a better understanding of the role of disulphide bridges and neighboring *N*-linked glycans in natively folded HIV-1 gp120, and to better insights in the effect of the introduction of the novel *N*-glycosylation sites on the 3D gp120 structure.

## Conclusions

We demonstrated that conserved *N*-linked glycans appear preferentially near, or in close proximity to, disulphide bridges in HIV-1 gp120, either at asparagines directly neighboring the involved cysteines or at asparagines located at the C-terminal side of the cysteine. The deletion of these *N*-glycans in some cases had significant detrimental effects on viral functions such as infectivity and transmission potential. On the other hand, introduction of *N*-glycans at positions in gp120 that seem to be disfavored for *N*-glycosylation (as evidenced by the statistically significant absence of glycosylation motifs at these amino acid positions) was also shown to result in loss of viral infectivity in some mutants. These data indicate that *N*-glycans are not spread randomly across gp120 and that their locations are of critical importance for the integrity of gp120 functions such as the infectivity and transmission potential of the virus.

## Supporting Information

S1 Fig Probability of finding an *N*-linked glycosylation site at a position 1–5 amino acids from a disulphide bridge in HCV E1 (A) and E2 (B). The amino acid sequences of both glycoproteins were obtained from NCBI (GenBank ID ABC40379.1 and NCBI Reference Sequence NP_751921.1, respectively). The allocation of *N*-glycosylation sites and disulphide bridges in E1 and E2 was based on publications of Wahid *et al*. [14] and Krey *et al*. [15] respectively. The graph shows the relative probabilities of a glycosylated asparagine at 1, 2, 3, 4 or 5 amino acid positions away from the cysteines involved in disulphide bridges. Negative amino acid positions correspond to positions at the N-terminal site of the cysteine, positive amino acid positions correspond to positions at the C-terminal site of the cysteine. The striped line indicates the probablities in case of random distribution of *N*-glycosylation sites.
(TIF)

S1 Table Conservation of cysteines involved in disulphide bridges in gp120. ^a^ Amino acid numbering based on the consensus of consensus HIV Env sequences obtained from the alignment of consensus sequences, available on the HIV sequence database website [11]. The cysteines are presented as pairs, corresponding with disulphide bond-associated cysteines in native HIV-1 gp120. The level of conservation over a series of 180 HIV-1 (group M) strains is shown for each cysteine. The HIV Env amino acid sequences of these HIV-1 strains were obtained from the HIV sequence compendium of 2014, available on the HIV sequence database [11].
(DOCX)

S2 Table Details on the probability calculations for the co-localization of disulphide bridges and *N*-glycosylation sites in HIV gp120. Negative amino acid positions correspond to positions at the N-terminal side away from a conserved cysteine, while positive amino acid positions correspond to positions at the C-terminal side away from a conserved cysteine. ^a^ The positions and levels of conservation of *N*-glycans of HIV-1 gp120 were obtained using the N-glycosite software available on the HIV sequence database website [11], based on an alignment of consensus sequences (2004) [11]. The alignment contained consensus sequences for HIV-1 subtypes A1, A2, B, C, D, F1, F2, G, H, CRF01-AE, CRF02-AG, CRF03-AB, CRF04-cpx, CRF06-cpx, CRF08-BC, CRF10-CD, CRF11-cpx, CRF12-BF, and CRF14-BG. The consensus of consensus sequences and the ancestral sequences were excluded from the analysis. ^b^ This number was obtained as follows: the consensus of consensus HIV Env sequences obtained from the alignment of consensus sequences [11] consists of a total of 518 amino acids, of which 18 are conserved cysteines and 18 positions are at the -2 position with respect of a conserved cysteine (= 2 amino acids upstream of a cysteine). In case of random distribution of *N*-glycans across gp120, a total of 482 amino acid positions would therefore be available (= 518 minus 18 minus 18). ^c^ The conserved cysteines 125 and 130 are separated by only 4 amino acids, implying that at the +5 position of cysteine 125 and at the -5 position of cysteine 130 no glycan can be present.
(DOCX)

S3 Table Oligonucleotide sequences of the primers used for site-directed mutagenesis and sequencing of gp120. (DOCX)

S4 Table Overview of the mutations introducted into HIV-1_NL4.3_ gp120. Amino acid numbering based on the HIV-1 strain HXB2 gp120. For the deletion of a disulphide bridge, one of the involved cysteines was mutated into an alanine. For the deletion of an *N*-linked glycan, the asparagine of the *N*-glycosylation site was mutated into a glutamine. In order to insert new *N*-glycosylation sites, an asparagine was introduced in combination with a serine two amino acid positions downstream of the newly-introduced asparagine. The insertion of an *N*-glycan at positions 291 and 292 was also combined with the simultaneous deletion of the N295 glycan, as indicated between parentheses.
(DOCX)
