# Supplementary figures and images for: Several N-Glycans on the HIV Envelope Glycoprotein gp120 Preferentially Locate Near Disulphide Bridges and Are Required for Efficient Infectivity and Virus Transmission
Source: PLoS One. 2015 Jun 29;10(6):e0130621. doi: 10.1371/journal.pone.0130621 (PMC4488071; doi:10.1371/journal.pone.0130621)

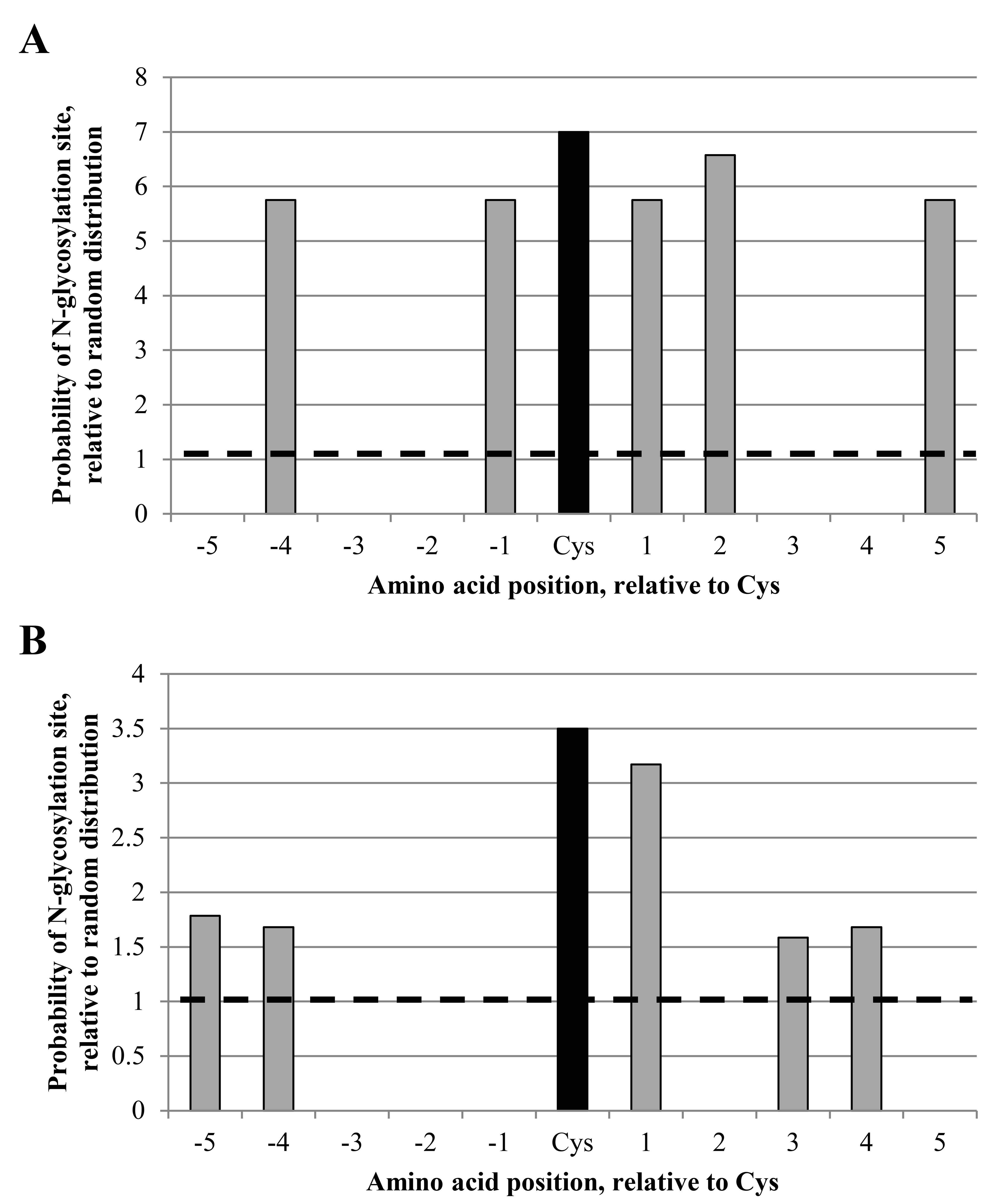

Supplement: S1 Fig — The amino acid sequences of both glycoproteins were obtained from NCBI (GenBank ID ABC40379.1 and NCBI Reference Sequence NP_751921.1, respectively). The allocation of N-glycosylation sites and disulphide bridges in E1 and E2 was based on publications of Wahid et al. [14] and Krey et al. [15] respectively. The graph shows the relative probabilities of a glycosylated asparagine at 1, 2, 3, 4 or 5 amino acid positions away from the cysteines involved in disulphide bridges. Negative amino acid positions correspond to positions at the N-terminal site of the cysteine, positive amino acid positions correspond to positions at the C-terminal site of the cysteine. The striped line indicates the probablities in case of random distribution of N-glycosylation sites. (TIF) [file pone.0130621.s001.tif]
