# Supplementary material for: Several N-Glycans on the HIV Envelope Glycoprotein gp120 Preferentially Locate Near Disulphide Bridges and Are Required for Efficient Infectivity and Virus Transmission
Source: PLoS One. 2015 Jun 29;10(6):e0130621. doi: 10.1371/journal.pone.0130621 (PMC4488071; doi:10.1371/journal.pone.0130621)
Supplement: S1 Table — a Amino acid numbering based on the consensus of consensus HIV Env sequences obtained from the alignment of consensus sequences, available on the HIV sequence database website [11]. The cysteines are presented as pairs, corresponding with disulphide bond-associated cysteines in native HIV-1 gp120. The level of conservation over a series of 180 HIV-1 (group M) strains is shown for each cysteine. The HIV Env amino acid sequences of these HIV-1 strains were obtained from the HIV sequence compendium of 2014, available on the HIV sequence database [11]. (DOCX) [file pone.0130621.s002.docx]

**S1 Table. Conservation of cysteines involved in disulphide bridges in gp120**

| **Disulphide (cysteine) positions in gp120 of consensus sequence^a^** | **Disulphide (cysteine) positions in gp120 of strain HIV-1_HXB2_** | **Cysteine conservation (%)** |
| --- | --- | --- |
| 53 | 54 | 100.0 |
| 73 | 74 | 100.0 |
| 118 | 119 | 100.0 |
| 214 | 205 | 99.4 |
| 125 | 126 | 99.4 |
| 205 | 196 | 98.9 |
| 130 | 131 | 99.4 |
| 160 | 157 | 100.0 |
| 227 | 218 | 100.0 |
| 256 | 247 | 98.9 |
| 237 | 228 | 99.4 |
| 248 | 239 | 99.4 |
| 305 | 296 | 100.0 |
| 339 | 331 | 98.9 |
| 385 | 378 | 98.3 |
| 451 | 445 | 99.4 |
| 392 | 385 | 100.0 |
| 424 | 418 | 98.3 |

^a^ Amino acid numbering based on the consensus of consensus HIV Env sequences obtained from the alignment of consensus sequences, available on the HIV sequence database website [11].

The cysteines are presented as pairs, corresponding with disulphide bond-associated cysteines in native HIV-1 gp120. The level of conservation over a series of 180 HIV-1 (group M) strains is shown for each cysteine. The HIV Env amino acid sequences of these HIV-1 strains were obtained from the HIV sequence compendium of 2014, available on the HIV sequence database [11].
