# Supplementary material for: Several N-Glycans on the HIV Envelope Glycoprotein gp120 Preferentially Locate Near Disulphide Bridges and Are Required for Efficient Infectivity and Virus Transmission
Source: PLoS One. 2015 Jun 29;10(6):e0130621. doi: 10.1371/journal.pone.0130621 (PMC4488071; doi:10.1371/journal.pone.0130621)
Supplement: S2 Table — Negative amino acid positions correspond to positions at the N-terminal side away from a conserved cysteine, while positive amino acid positions correspond to positions at the C-terminal side away from a conserved cysteine. a The positions and levels of conservation of N-glycans of HIV-1 gp120 were obtained using the N-glycosite software available on the HIV sequence database website [11], based on an alignment of consensus sequences (2004) [11]. The alignment contained consensus sequences for HIV-1 subtypes A1, A2, B, C, D, F1, F2, G, H, CRF01-AE, CRF02-AG, CRF03-AB, CRF04-cpx, CRF06-cpx, CRF08-BC, CRF10-CD, CRF11-cpx, CRF12-BF, and CRF14-BG. The consensus of consensus sequences and the ancestral sequences were excluded from the analysis. b This number was obtained as follows: the consensus of consensus HIV Env sequences obtained from the alignment of consensus sequences [11] consists of a total of 518 amino acids, of which 18 are conserved cysteines and 18 positions are at the -2 position with respect of a conserved cysteine (= 2 amino acids upstream of a cysteine). In case of random distribution of N-glycans across gp120, a total of 482 amino acid positions would therefore be available (= 518 minus 18 minus 18). c The conserved cysteines 125 and 130 are separated by only 4 amino acids, implying that at the +5 position of cysteine 125 and at the -5 position of cysteine 130 no glycan can be present. (DOCX) [file pone.0130621.s003.docx]

**S2 Table. Details on the probability calculations for the co-localization of disulphide bridges and *N*-glycosylation sites in HIV gp120**

|  | **Conserved glycans**  **(> 50% conserved)** | **Non-conserved glycans**  **(< 50% conserved)** |
| --- | --- | --- |
| Number of conserved cysteines, which are involved in disulphide bridges in gp120 | 18 | |
| **Calculation of the random chance of finding an *N*-glycan at a position 1, 2, 3, 4 or 5 amino acids away from a cysteine involved in a disulphide bridge in gp120, either at the N-terminal or the C-terminal side of the cysteine** | | |
| Number of *N*-glycans in gp120^a^ | 24 | 33 |
| Number of amino acid positions in gp120 that could, in theory, be glycosylated^b^ | 482 | 482 |
| Chance of a glycan at one specific position within gp120 | 24/482 = 0.05 | 33/482 = 0.07 |
| Chance of finding a glycan at a position 1, 2, 3 or 4 amino acids away from a cysteine, either at the N-terminal or the C-terminal side of the cysteine | 18*0.05 = 0.90 | 18*0.07 = 1.23 |
| Chance of finding a glycan at a position 5 amino acids away from a cysteine, either at the N-terminal or the C-terminal side of the cysteine^c^ | 17*0.05 = 0.85 | 17*0.07 = 1.16 |
| **Actual number of *N*-glycans at a position 1, 2, 3, 4 or 5 amino acids away from a cysteine involved in a disulphide bridge in gp120** | | |
| Number of glycans at position -5 away from a cysteine | 0 | 1 |
| Number of glycans at position -4 away from a cysteine | 0 | 0 |
| Number of glycans at position -3 away from a cysteine | 0 | 0 |
| Number of glycans at position -2 away from a cysteine | 0 | 0 |
| Number of glycans at position -1 away from a cysteine | 3 | 1 |
| Number of glycans at position +1 away from a cysteine | 3 | 0 |
| Number of glycans at position +2 away from a cysteine | 1 | 1 |
| Number of glycans at position +3 away from a cysteine | 2 | 1 |
| Number of glycans at position +4 away from a cysteine | 0 | 3 |
| Number of glycans at position +5 away from a cysteine | 1 | 0 |
| **Probability of finding an *N-*glycan at a position 1, 2, 3, 4 or 5 amino acids away from a cysteine involved in a disulphide bridge in gp120, relative to the probability in case of random distribution of *N*-glycans over gp120** | | |
| Probability of a glycan at a position -5 away from a cysteine | 0 / 0.85 = 0 | 1 / 1.16 = 0.9 |
| Probability of a glycan at a position -4 away from a cysteine | 0 / 0.90 = 0 | 0 / 1.23 = 0 |
| Probability of a glycan at a position -3 away from a cysteine | 0 / 0.90 = 0 | 0 / 1.23 = 0 |
| Probability of a glycan at a position -2 away from a cysteine | 0 / 0.90 = 0 | 0 / 1.23 = 0 |
| Probability of a glycan at a position -1 away from a cysteine | 3 / 0.90 = 3.3 | 1 / 1.23 = 0.8 |
| Probability of a glycan at a position +1 away from a cysteine | 3 / 0.90 = 3.3 | 0 / 1.23 = 0 |
| Probability of a glycan at a position +2 away from a cysteine | 1 / 0.90 = 1.1 | 1 / 1.23 = 0.8 |
| Probability of a glycan at a position +3 away from a cysteine | 2 / 0.90 = 2.2 | 1 / 1.23 = 0.8 |
| Probability of a glycan at a position +4 away from a cysteine | 0 / 0.90 = 0 | 3 / 1.23 = 2.4 |
| Probability of a glycan at a position +5 away from a cysteine | 1 / 0.85 = 1.2 | 0 / 1.16 = 0 |

Negative amino acid positions correspond to positions at the N-terminal side away from a conserved cysteine, while positive amino acid positions correspond to positions at the C-terminal side away from a conserved cysteine.

^a^ The positions and levels of conservation of *N*-glycans of HIV-1 gp120 were obtained using the N-glycosite software available on the HIV sequence database website [11], based on an alignment of consensus sequences (2004) [11]. The alignment contained consensus sequences for HIV-1 subtypes A1, A2, B, C, D, F1, F2, G, H, CRF01-AE, CRF02-AG, CRF03-AB, CRF04-cpx, CRF06-cpx, CRF08-BC, CRF10-CD, CRF11-cpx, CRF12-BF, and CRF14-BG. The consensus of consensus sequences and the ancestral sequences were excluded from the analysis.

^b^ This number was obtained as follows: the consensus of consensus HIV Env sequences obtained from the alignment of consensus sequences [11] consists of a total of 518 amino acids, of which 18 are conserved cysteines and 18 positions are at the -2 position with respect of a conserved cysteine (= 2 amino acids upstream of a cysteine). In case of random distribution of *N*-glycans across gp120, a total of 482 amino acid positions would therefore be available (= 518 minus 18 minus 18).

^c^ The conserved cysteines 125 and 130 are separated by only 4 amino acids, implying that at the +5 position of cysteine 125 and at the -5 position of cysteine 130 no glycan can be present.
