# Supplementary material for: Several N-Glycans on the HIV Envelope Glycoprotein gp120 Preferentially Locate Near Disulphide Bridges and Are Required for Efficient Infectivity and Virus Transmission
Source: PLoS One. 2015 Jun 29;10(6):e0130621. doi: 10.1371/journal.pone.0130621 (PMC4488071; doi:10.1371/journal.pone.0130621)
Supplement: S3 Table — (DOCX) [file pone.0130621.s004.docx]

**S3 Table. Oligonucleotide sequences of the primers used for site-directed mutagenesis and sequencing of gp120.**

| **Primer** | **Nucleotide sequence** | **Description** |
| --- | --- | --- |
| Mut_C157A_F | GGA GAG ATA AAA AAC GCC TCT TTC AAT ATC | Forward mutagenesis primer for C157A |
| Mut_C157A_R | GAT ATT GAA AGA GGC GTT TTT TAT CTC TCC | Reverse mutagenesis primer for C157A |
| Mut_C196A_F | GCTATAGGTTGATAAGTGCTAACACCTCAGTCATTAC | Forward mutagenesis primer for C196A |
| Mut_C196A_R | GTA ATG ACT GAG GTG TTA GCA CTT ATC AAC CTA TAG C | Reverse mutagenesis primer for C196A |
| Mut_C228A_F | GTT TTG CGA TTC TAA AAG CTA ATA ATA AGA CGT TC | Forward mutagenesis primer for C228A |
| Mut_C228A_R | GAA CGT CTT ATT ATT AGC TTT TAG AAT CGC AAA AC | Reverse mutagenesis primer for C228A |
| Mut_C331A_F | GAG ACA AGC ACA TGC TAA CAT TAG TAG AGC | Forward mutagenesis primer for C331A |
| Mut_C331A_R | GCT CTA CTA ATG TTA GCA TGT GCT TGT CTC | Reverse mutagenesis primer for C331A |
| Mut_C385A_F | GGGAATTTTTCTACGCTAATTCAACACAACTG | Forward mutagenesis primer for C385A |
| Mut_C385A_R | CAG TTG TGT TGA ATT AGC GTA GAA AAA TTC CC | Reverse mutagenesis primer for C385A |
| Mut_N156Q_F | GAA AGG AGA GAT AAA ACA ATG CTC TTT CAA TAT C | Forward mutagenesis primer for N156Q |
| Mut_N156Q_R | GAT ATT GAA AGA GCA TTG TTT TAT CTC TCC TTT C | Reverse mutagenesis primer for N156Q |
| Mut_N197Q_F | GCTATAGGTTGATAAGTTGTCAGACCTCAGTCATTACACAG | Forward mutagenesis primer for N197Q |
| Mut_N197Q_R | CTG TGT AAT GAC TGA GGT CTG ACA ACT TAT CAA CCT ATA GC | Reverse mutagenesis primer for N197Q |
| Mut_N230Q_F | GCG ATT CTA AAA TGT AAT CAG AAG ACG TTC AAT GGA AC | Forward mutagenesis primer for N230Q |
| Mut_N230Q_R | GTT CCA TTG AAC GTC TTC TGA TTA CAT TTT AGA ATC GC | Reverse mutagenesis primer for N230Q |
| Mut_N241Q_F | GGA ACA GGA CCA TGT ACA CAG GTC AGC ACA GTA CAA TG | Forward mutagenesis primer for N241Q |
| Mut_N241Q_R | CAT TGT ACT GTG CTG ACC TGT GTA CAT GGT CCT GTT CC | Reverse mutagenesis primer for N241Q |
| Mut_N295Q_F | CAC ATC TGT AGA AAT TCA GTG TAC AAG ACC CAA C | Forward mutagenesis primer for N295Q |
| Mut_N295Q_R | GTT GGG TCT TGT ACA CTG AAT TTC TAC AGA TGT G | Reverse mutagenesis primer for N295Q |
| Mut_N332Q_F | GAG ACA AGC ACA TTG TCA GAT TAG TAG AGC AAA ATG G | Forward mutagenesis primer for N332Q |
| Mut_N332Q_R | CCA TTT TGC TCT ACT AAT CTG ACA ATG TGC TTG TCT C | Reverse mutagenesis primer for N332Q |
| Mut_N386Q_F | GGGAATTTTTCTACTGTCAATCAACACAACTGTTTAATAGTAC | Forward mutagenesis primer for N386Q |
| Mut_N386Q_R | GTA CTA TTA AACA GTT GTG TTG ATT GAC AGT AGA AAA ATT CCC | Reverse mutagenesis primer for N386Q |
| Mut_V292N/I294S_F | CTG AAC ACA TCT AAT GAA TCT AAT TGT ACA AGA | Forward mutagenesis primer for V292N/I294S |
| Mut_V292N/I294S_R | TCT TGT ACA ATT AGA TTC ATT AGA TGT GTT CAG | Reverse mutagenesis primer for V292N/I294S |
| Mut_S291N/E293S_F | CAG CTG AAC ACA AAT GTA TCT ATT AAT TGT ACA | Forward mutagenesis primer for S291N/E293S |
| Mut_S291N/E293S_R | TGT ACA ATT AAT AGA TAC ATT TGT GTT CAG CTG | Reverse mutagenesis primer for S291N/E293S |
| Mut_E293N/N295S_F | AAC ACA TCT GTA AAT ATT TCT TGT ACA AGA CCC | Forward mutagenesis primer for E293N/N295S |
| Mut_E293N/N295S_R | GGG TCT TGT ACA AGA AAT ATT TAC AGA TGT GTT | Reverse mutagenesis primer for E293N/N295S |
| Mut_V292N/I294S/N295Q_F | CTG AAC ACA TCT AAT GAA TCT CAG TGT ACA AGA CCC AAC | Forward mutagenesis primer for V292N/I294S/N295Q |
| Mut_V292N/I294S/N295Q_R | GTT GGG TCT TGT ACA CTG AGA TTC ATT AGA TGT GTT CAG | Reverse mutagenesis primer for V292N/I294S/N295Q |
| Mut_S291N/E293S/N295Q_F | CAG CTG AAC ACA AAT GTA TCT ATT CAG TGT ACA AGA CCC | Forward mutagenesis primer for S291N/E293S/N295Q |
| Mut_S291N/E293S/N295Q_R | GGG TCT TGT ACA CTG AAT AGA TAC ATT TGT GTT CAG CTG | Reverse mutagenesis primer for S291N/E293S/N295Q |
| Mut_I294S_F | CAC ATC TGT AGA ATC TAA TTG TAC AAG A | Forward mutagenesis primer for I294S |
| Mut_I294S_R | TCT TGT ACA ATT AGA TTC TAC AGA TGT G | Reverse mutagenesis primer for I294S |
| Mut_V292N_F | CTG AAC ACA TCT AAT GAA ATT AAT TGT | Forward mutagenesis primer for V292N |
| Mut_V292N_R | ACA ATT AAT TTC ATT AGA TGT GTT CAG | Reverse mutagenesis primer for V292N |
| Mut_F382N/Y384S_F | AAT TGT GGA GGG GAA AAT TTC TCA TGT AAT TCA ACA C | Forward mutagenesis primer for F382N/Y384S |
| Mut_F382N/Y384S_R | GTG TTG AAT TAC ATG AGA AAT TTT CCC CTC CAC AAT T | Reverse mutagenesis primer for F382N/Y384S |
| Mut_E381N/F383S_F | TTT AAT TGT GGA GGG AAT TTT TCA TAC TGT AAT TCA AC | Forward mutagenesis primer for E381N/F383S |
| Mut_E381N/F383S_R | GTT GAA TTA CAG TAT GAA AAA TTC CCT CCA CAA TTA AA | Reverse mutagenesis primer for E381N/F383S |
| Mut_G380N/F382S_F | GTT TTA ATT GTG GAA ATG AAT CAT TCT ACT GTA ATT C | Forward mutagenesis primer for G380N/F382S |
| Mut_G380N/F382S_R | GAA TTA CAG TAG AAT GAT TCA TTT CCA CAA TTA AAA C | Reverse mutagenesis primer for G380N/F380S |
| AV302 [18] | CTAATAGAAAGAGCAGAAGACAGTGG | Forward sequencing primer |
| AV304 [18] | ACATGTGGAAAAATGACATGGT | Forward sequencing primer |
| AV305 [18] | GAGTGGGGTTAATTTTACACATGG | Reverse sequencing primer |
| AV306 [18] | TGTCAGCACAGTACAATGTACACA | Forward sequencing primer |
| AV307 [18] | TCTTCTTCTGCTAGACTGCCAT | Reverse sequencing primer |
| AV308 [18] | TCCTCAGGAGGGGACCCAGAAATT | Forward sequencing primer |
| AV309 [18] | CARTAGAAAAATTCYCCTCYACA | Reverse sequencing primer |
| AV313 [18] | TCCYTCATATYTCCTCCTCCAGGTC | Reverse sequencing primer |
