# Supplementary material for: Several N-Glycans on the HIV Envelope Glycoprotein gp120 Preferentially Locate Near Disulphide Bridges and Are Required for Efficient Infectivity and Virus Transmission
Source: PLoS One. 2015 Jun 29;10(6):e0130621. doi: 10.1371/journal.pone.0130621 (PMC4488071; doi:10.1371/journal.pone.0130621)
Supplement: S4 Table — Amino acid numbering based on the HIV-1 strain HXB2 gp120. For the deletion of a disulphide bridge, one of the involved cysteines was mutated into an alanine. For the deletion of an N-linked glycan, the asparagine of the N-glycosylation site was mutated into a glutamine. In order to insert new N-glycosylation sites, an asparagine was introduced in combination with a serine two amino acid positions downstream of the newly-introduced asparagine. The insertion of an N-glycan at positions 291 and 292 was also combined with the simultaneous deletion of the N295 glycan, as indicated between parentheses. (DOCX) [file pone.0130621.s005.docx]

**S4 Table. Overview of the mutations introducted into HIV-1_NL4.3_ gp120**

| **Mutated disulphide-engaged cysteine** | **Deleted *N*-linked glycosylation site** | **Inserted *N*-linked glycosylation site** |
| --- | --- | --- |
| C157A | N156Q | S291N + E293S (+ N295Q) |
| C196A | N197Q | V292N + I294S (+ N295Q) |
| C228A | N230Q | E293N + N295S |
| C331A | N241Q | G380N + F382S |
| C385A | N295Q | E381N + F383S |
|  | N332Q | F382N + Y384S |
|  | N386Q |  |

Amino acid numbering based on the HIV-1 strain HXB2 gp120.

For the deletion of a disulphide bridge, one of the involved cysteines was mutated into an alanine. For the deletion of an *N*-linked glycan, the asparagine of the *N*-glycosylation site was mutated into a glutamine. In order to insert new *N-*glycosylation sites, an asparagine was introduced in combination with a serine two amino acid positions downstream of the newly-introduced asparagine. The insertion of an *N*-glycan at positions 291 and 292 was also combined with the simultaneous deletion of the N295 glycan, as indicated between parentheses.
